# A major role of coumarin-dependent ferric iron reduction in strategy I-type iron acquisition in Arabidopsis

**DOI:** 10.1093/plcell/koad279

**Published:** 2023-11-28

**Authors:** Vanessa Paffrath, Yudelsy A Tandron Moya, Günther Weber, Nicolaus von Wirén, Ricardo F H Giehl

**Affiliations:** Leibniz Institute of Plant Genetics and Crop Plant Research (IPK) OT Gatersleben, Corrensstr 3, 06466 Seeland, Germany; Leibniz Institute of Plant Genetics and Crop Plant Research (IPK) OT Gatersleben, Corrensstr 3, 06466 Seeland, Germany; Leibniz-Institut für Analytische Wissenschaften (ISAS) e.V., Bunsen-Kirchhoff-Str 11, 44139 Dortmund, Germany; Leibniz Institute of Plant Genetics and Crop Plant Research (IPK) OT Gatersleben, Corrensstr 3, 06466 Seeland, Germany; Leibniz Institute of Plant Genetics and Crop Plant Research (IPK) OT Gatersleben, Corrensstr 3, 06466 Seeland, Germany

## Abstract

Many non-graminaceous species release various coumarins in response to iron (Fe) deficiency. However, the physiological relevance of these coumarins remains poorly understood. Here, we show that the three enzymes leading to sideretin biosynthesis co-exist in Arabidopsis (*Arabidopsis thaliana*) epidermal and cortical cells and that the shift to fraxetin at alkaline pH depends on MYB72-mediated repression of *CYTOCHROME P450*, *FAMILY 82*, *SUBFAMILY C*, *POLYPEPTIDE 4* (*CYP82C4*). In vitro, only fraxetin and sideretin can reduce part of the Fe(III) that they mobilize. We demonstrate that coumarin-mediated Fe(III) reduction is critical under acidic conditions, as fraxetin and sideretin can complement the Fe(III)-chelate reductase mutant *ferric reduction oxidase 2* (*fro2*), and disruption of coumarin biosynthesis in *fro2* plants impairs Fe acquisition similar to in the Fe(II) uptake-deficient mutant *iron-regulated transporter 1* (*irt1*). Disruption of sideretin biosynthesis in a *fro2 cyp82C4-1* double mutant revealed that sideretin is the dominant chemical reductant that functions with FRO2 to mediate Fe(II) formation for root uptake. At alkaline pH, Fe(III) reduction by coumarins becomes almost negligible but fraxetin still sustains high Fe(III) mobilization, suggesting that its main function is to provide chelated Fe(III) for FRO2. Our study indicates that strategy-I plants link sideretin and fraxetin biosynthesis and secretion to external pH to recruit distinct coumarin chemical activities to maximize Fe acquisition according to prevailing soil pH conditions.

## Teaser

Environmental pH-dependent regulation of coumarin synthesis recruits the superior ferric iron reductant (sideretin) in acidic conditions and the most efficient chelator (fraxetin) at alkaline pH.

**IN A NUTSHELL**

**Background:** Although iron (Fe) is abundant in the Earth's crust, the availability of this micronutrient to plants is decreased under slightly acidic to alkaline pH conditions. Non-graminaceous species, such as Arabidopsis (*Arabidopsis thaliana*), rely on the “strategy I” Fe acquisition mechanism, which requires the reduction of Fe(III) prior to uptake. These plants are typically more prone to high pH-induced Fe deficiency because Fe(III) reductase activity is pH sensitive, such as that of FERRIC REDUCTION OXIDASE2 (FRO2). Iron acquisition in species employing the reduction-based strategy is often supported by the release of coumarins, secondary metabolites derived from the phenylpropanoid pathway.

**Questions:** Do the redox-active coumarins secreted by Fe-deficient Arabidopsis plants contribute to ferric Fe reduction in roots? If so, how is this function linked to FRO2? How are coumarin composition and the chemical activities of distinct coumarins modulated by environmental pH?

**Findings:** With chemical complementation experiments and by disrupting single steps of the coumarin biosynthesis pathway in the *fro2* mutant, we revealed that coumarins play a physiologically relevant role in Fe(III) reduction. We demonstrated that sideretin is the dominant chemical reductant in Arabidopsis and, with FRO2, contributes to generating Fe(II) for root uptake under acidic conditions. At alkaline pH, the transcription factor MYB72 shifts coumarin biosynthesis away from sideretin and toward fraxetin. Under these conditions, instead of ferric Fe reduction, the predominant function of fraxetin is to provide soluble Fe(III) substrates for FRO2.

**Next steps:** Future studies are needed to determine whether there is genetic variability for coumarin-mediated ferric Fe reduction and to characterize the putative role of MYB72 in pH-dependent modulation of coumarin composition. Furthermore, it will be of interest to investigate whether and how coumarin-mediated Fe(III) reduction affects the root microbiota.

## Introduction

Iron (Fe) is a micronutrient with key functions in many biological processes. In plants, insufficient Fe uptake impairs growth, decreases fitness and, in crop species, leads to loss of yield and quality (Briat et al. 2015). In most environments, Fe deficiency is not caused by low total Fe concentrations in soils but by low Fe bioavailability. Under slightly acidic to alkaline pH conditions and sufficient aeration, common to most soils, Fe is mostly present as Fe(III), i.e. in its ferric form, contained in poorly soluble Fe-bearing minerals (Lindsay and Schwab 1982; Lemanceau et al. 2009). Consequently, the solubility and dissolution (i.e. mobilization) rate of Fe(III) (hydr)oxides determine how much Fe becomes available for plants. Mobilization from sparingly available Fe sources can be facilitated by proton-promoted, ligand-controlled, or reductive dissolution processes (Schwertmann 1991; Kraemer 2004). To actively extract Fe from the soil, plants have evolved Fe acquisition strategies that exploit one or more of these processes.

Plant species from the Poaceae family (grasses) have evolved a Fe acquisition strategy centered on Fe chelation by synthesizing and secreting mugineic acid-type phytosiderophores, which are small Fe-complexing ligands derived from nicotianamine (Römheld and Marschner 1986; Shojima et al. 1990; Ma and Nomoto 1993; Higuchi et al. 1999). The phytosiderophores produced by grasses bind Fe(III) in a hexadentate fashion via their amino and carboxyl groups, forming soluble 1:1 complexes (Mino et al. 1983). Grass roots can then take up intact Fe(III)-phytosiderophore complexes by specific transporters, such as YELLOW STRIPE 1 (YS1), present at the plasma membrane of root cells (von Wirén et al. 1994; Curie et al. 2001; Schaaf et al. 2004). In all non-graminaceous plants, Fe is imported into root cells as free Fe^2+^ ion (Eide et al. 1996). Thus, efficient reduction of Fe(III) prior to uptake is a critical step to maintain sufficient Fe uptake in these plant species.

Specialized membrane-bound ferric reductases, such as FERRIC REDUCTION OXIDASE2 (FRO2) in Arabidopsis (*Arabidopsis thaliana*) (Robinson et al. 1999), are responsible for a large part of the Fe(III) reduction taking place in roots. The generated Fe^2+^ ion can then be taken up by IRON REGULATED TRANSPORTER1 (IRT1), the main root Fe uptake transporter (Henriques et al. 2002; Vert et al. 2002). However, being bound to the plasma membrane of root cells, FRO-mediated Fe(III) reduction per se is limited spatially to soluble Fe(III)-complexes reaching the membrane surface. Fe(III) solubilization is further facilitated by localized acidification of the root apoplast and surrounding rhizosphere via proton extrusion mediated by P-type ATPases, such as PLASMA MEMBRANE PROTON ATPASE 2 (AHA2) (Santi and Schmidt 2009). The secreted protons help to solubilize precipitated soil Fe (Schwertmann 1991) and to maintain the apoplastic pH at a level that favors optimal FRO activity (Susin et al. 1996). However, Fe dissolution by protonation is comparatively slow and ferric Fe solubilized by this mechanism may re-precipitate if not immediately chelated (Robin et al. 2008).

**Figure 1.**
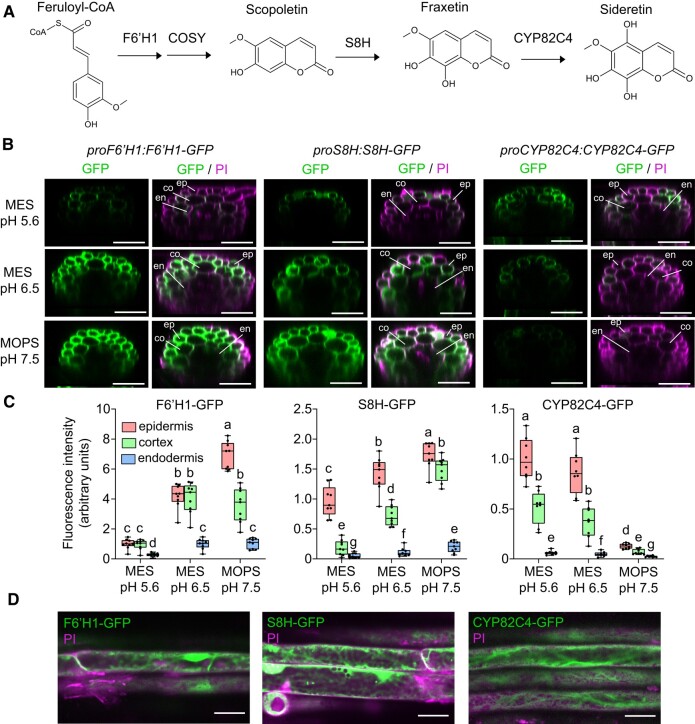
pH-dependent abundance and tissue-specific localization of F6′H1, S8H, and CYP82C4 in roots exposed to different external pH and buffer conditions in the absence of Fe. **A)** Simplified coumarin biosynthesis pathway. F6′H1 converts feruloyl-CoA derived from the phenylpropanoid pathway into 6′-hydroxyferuloyl-CoA, which is then converted to scopoletin partially spontaneously and by the activity of COSY. S8H and CYP82C4 generate the catecholic coumarins fraxetin and sideretin, respectively. Only the aglycones of these molecules are drawn for simplicity. **B and C)** GFP and merged GFP and propidium iodide (PI) signals detected in the root hair zone of *proF6′H1:F6′H1-GFP*, *proS8H:S8H-GFP*, and *proCYP82C4:CYP82C4-GFP* translation fusion lines. Plants were precultured for 10 d on half-strength Murashige and Skoog (MS) medium with 40 *µ*M Fe-EDTA at pH 5.6 and then transferred to half-strength MS medium without added Fe and buffered with either MES to pH 5.6 or pH 6.5, or MOPS to pH 7.5. Images were taken 4 d after transfer to the indicated conditions. Shown are transverse sections reconstituted from Z-stacks **(B)** and quantification of signals in epidermal, cortical, and endodermal cells **(C)**. Boxplots show the first quartile, median quartile, and third quartile. The whiskers extend to the minimum and maximum values (*n* = 8 independent roots for CYP82C4-GFP and *n* = 9 independent roots for F6′H1-GFP and S8H-GFP). Different letters indicate significant differences (*P* < 0.05) according to one-way ANOVA on ranks with post hoc Student–Newman–Keuls test. Scale bars, 50 *µ*m. Root tissue layers are labeled as ep: epidermis, co: cortex, and en: endodermis. PI, propidium iodide. **D)** Subcellular localization of F6′H1-GFP, S8H-GFP, and CYP82C4-GFP. Images were taken 4 d after transfer to half-strength MS medium without added Fe and buffered with MES to either pH 5.6 (CYP82C4-GFP) or pH 6.5 (F6′H1-GFP and S8H-GFP). Scale bars, 20 *µ*m.

While non-grasses do not produce mugineic acid-type phytosiderophores, they synthesize and exude different types of low-molecular-weight molecules, such as organic acids, flavins, and phenolics, which can help to mobilize Fe in the rhizosphere and root apoplast (Chai and Schachtman 2022). In Brassicaceae and several other plant families, Fe deficiency induces the secretion of several coumarins, which are phenolic compounds derived from the phenylpropanoid pathway (Fourcroy et al. 2014; Schmid et al. 2014; Schmidt et al. 2014; Sisó-Terraza et al. 2016; Rajniak et al. 2018; Siwinska et al. 2018; Tsai et al. 2018). The first committed step in coumarin biosynthesis is the *ortho*-hydroxylation of feruloyl-CoA catalyzed by the 2-oxoglutarate-dependent dioxygenase FERULOYL-CoA 6′-HYDROXYLASE 1 (F6′H1) (Fig. 1A; Kai et al. 2008). The generated 6-hydroxyferuloyl-CoA can then be converted to scopoletin by subsequent trans-cis isomerization and lactonization. These reactions can occur partially spontaneously—if tissues are exposed to light—or catalyzed enzymatically by the BAHD acyltransferase COUMARIN SYNTHASE (COSY) in light-protected organs (Vanholme et al. 2019). Scopoletin is then further converted into fraxetin and sideretin. Fraxetin is synthesized from scopoletin via a hydroxylation step catalyzed by the 2-oxoglutarate-dependent dioxygenase SCOPOLETIN 8-HYDROXYLASE (S8H) (Rajniak et al. 2018; Siwinska et al. 2018; Tsai et al. 2018). Then, in a second hydroxylation step, the cytochrome P450 enzyme CYP82C4 converts fraxetin into sideretin (Rajniak et al. 2018).

It has been shown that root-borne coumarins can directly contribute to Fe acquisition but still little is known about the relevance of their structural variability. Esculetin, fraxetin, and sideretin, which possess a catechol moiety (i.e. a benzene ring with 2 adjacent hydroxyl groups), are able to mobilize Fe from freshly precipitated Fe-hydroxides in vitro (Schmid et al. 2014; Sisó-Terraza et al. 2016; Rajniak et al. 2018). Fe mobilization by these coumarins is likely facilitated by chelation of ferric Fe by the catechol group, reminiscent of the mode of action of catechol-type bacterial siderophores (Hider and Kong 2010). Consequently, Fe(III) complexed with coumarins becomes available for enzymatic reduction at the plasma membrane. Indeed, coumarin deficiency resulting from *F6′H1* disruption causes Fe deficiency if plants grow on a poorly soluble Fe source (Rodriguez-Celma et al. 2013; Schmid et al. 2014). Coumarins may also reduce Fe(III), thereby potentially providing Fe^2+^ directly to IRT1. At least in vitro, some coumarins have been shown to reduce ferric Fe (Mladěnka et al. 2010; Schmidt et al. 2014; Sisó-Terraza et al. 2016; Rajniak et al. 2018; Tsai et al. 2018), but which coumarins efficiently reduce ferric Fe and what is the biological relevance of this process under different pH conditions remain unresolved. A previous study reported that supplying wild-type root exudates to a *fro2* mutant grown in hydroponics in the presence of poorly available Fe at pH 7.5 was unable to alleviate the Fe-deficiency symptoms of the mutant (Fourcroy et al. 2016). This was seen as indication for the inability of root exudates to confer sufficient reduction capacity and to bypass FRO2 for sustained Fe nutrition, at least at alkaline pH, as the effect was not assessed at other pH conditions. The activity of root-bound ferric reductases is pH sensitive and decreases at high pH (Romera et al. 1992; Susin et al. 1996). Soil pH may also determine the mode of action of coumarins. Previous studies have shown that the external pH can modulate the coumarin composition of root exudates (Sisó-Terraza et al. 2016; Rajniak et al. 2018; Gautam et al. 2021), which may contribute to the superior tolerance to elevated soil carbonate of certain small local populations of *A. thaliana* (Terés et al. 2019). However, to date, it has remained unclear whether and under which conditions Fe(III) reduction by coumarins plays a role in Fe acquisition, and whether coumarins may compensate for a loss of FRO2-mediated Fe acquisition.

The present study set out to determine the physiological relevance of coumarin-mediated Fe(III) reduction and its molecular regulation in response to external pH. By mutating single steps of coumarin biosynthesis in the *fro2* background, we unravel the contribution of individual coumarin species to reductive Fe acquisition. Furthermore, by combining in vitro assays with chemical complementation experiments, we demonstrate that FRO2 and coumarins play complementary roles in ferric Fe reduction at acidic pH, with a dominant role for sideretin. At alkaline conditions, coumarin-mediated ferric Fe reduction becomes less relevant and the predominant function of fraxetin, as the main catecholic coumarin released under these conditions, is to mobilize sparingly available ferric Fe for further reduction by FRO2.

## Results

### Tissue-specific localization and abundance of F6′H1, S8H, and CYP82C4 proteins are determined by external pH

In order to determine the cell type-specific localization and pH-dependency of the major steps of coumarin biosynthesis in *A. thaliana* roots, we generated transgenic lines expressing translational fusions of F6′H1-GFP, S8H-GFP, and CYP82C4-GFP under the control of the respective native promoters. Under Fe-depleted conditions, which induced shoot chlorosis irrespective of the external pH (Supplemental Fig. S1, A and B), F6′H1 protein was detected in epidermal and cortical cells under all pH conditions while S8H expanded from almost exclusive presence in the epidermis at pH 5.6 also to the cortex at pH 6.5 and, especially, at pH 7.5 (Fig. 1, B and C). CYP82C4, in turn, was more abundant in epidermal cells with cortical cells exhibiting only approximately half of CYP82C4-GFP-derived fluorescence at pH 5.6 and 6.5. The most dramatic pH-dependent change was observed for CYP82C4, whose accumulation was almost completely abolished at pH 7.5 (Figure 1, B and C). The negative effect of alkaline conditions on CYP82C4 displayed also at the transcriptional level (Supplemental Fig. S1C), in agreement with findings from recent studies (Tsai and Schmidt 2020; Gautam et al. 2021). At the subcellular level, we found that all three proteins were predominantly located in the cytosol with S8H-GFP occasionally exhibiting a punctate localization which could not yet be fully determined (Fig. 1D). A comparison to plants cultivated under different external pH in the presence of poorly soluble Fe indicated that cell type-specific localization of the three enzymes was not subject to Fe-dependent regulation (Supplemental Fig. S2).

### External pH affects the composition of coumarins in root extracts and exudates

**Figure 2.**
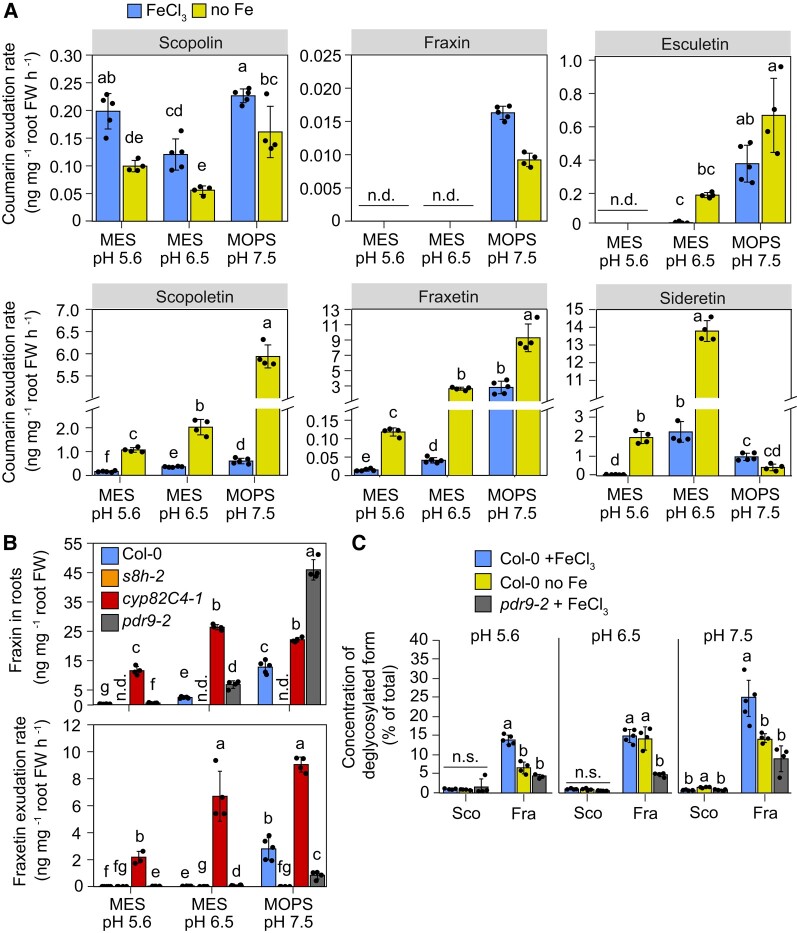
Effect of Fe, external pH, and coumarin export on coumarin concentrations in roots and exudates. **A)** Secretion rates of individual coumarin species in *A. thaliana* (Col-0) plants grown for 4 d under different Fe-limiting conditions and external pHs. Root exudates were collected for 6 h in MES- or MOPS-buffered water adjusted to the respective pH. Sideretin represents the sum of oxidized and reduced forms. Bars represent means ± Sd (*n* = 4 to 5 biological replicates composed of pooled exudates collected from 140 plants each). **B)** Concentration of fraxin in root extracts and secretion rate of fraxetin in wild-type (Col-0), *s8h-2*, *cyp82C4-1*, and *pdr9-2* plants grown for 4 d on half-strength Murashige and Skoog (MS) medium with 20 *µ*M FeCl_3_ (FeCl_3_). Bars represent means ± Sd (*n* = 4 to 5 biological replicates composed of pooled roots of 140 plants each). **C)** Percentage of the deglycosylated form for the indicated coumarin pairs in root extracts of wild-type (Col-0) and *pdr9-2* plants grown for 4 d on the indicated pH and Fe conditions. Bars represent means ± Sd (*n* = 4 to 5 biological replicates composed of pooled exudates collected from 140 plants each). Sco, scopolin/scopoletin pair; Fra, fraxin/fraxetin pair. FW, fresh weight; n.d., not detected. In **A to C**, plants were precultured for 10 d on half-strength MS medium with 40 *µ*M Fe-EDTA at pH 5.6 and then transferred to half-strength MS medium with 20 *µ*M FeCl_3_ (FeCl_3_) or without added Fe and 15 *µ*M ferrozine (no Fe) buffered with MES to pH 5.6 or pH 6.5, or MOPS to pH 7.5. Different letters indicate significant differences (*P* < 0.05) among all groups **(A and B)** or within each metabolite group **(C)** according to one-way ANOVA with post hoc Tukey's test (scopolin and data in **(C)** or one-way ANOVA on ranks with post hoc Student–Newman–Keuls test (all other comparisons). n.s., not significant (*P* > 0.05) according to one-way ANOVA.

Considering that pH-dependent changes in the expression of coumarin-related genes is also modulated by Fe (Gautam et al. 2021), we compared the coumarin composition of root exudates in wild-type plants grown at increasing external pH without supplied Fe or in presence of poorly soluble FeCl_3_. Following the expected increase in Fe precipitation with increasing medium pH, the shoot Fe status of the plants declined (Supplemental Fig. S3A). We then quantified the concentration of different coumarins in root extracts or exudates with liquid chromatography-mass spectrometry by using authentic coumarin standards. In agreement with previous studies (Sisó-Terraza et al. 2016; Rajniak et al. 2018; Gautam et al. 2021) at low pH, Fe starvation significantly increased secretion of scopoletin, fraxetin, and sideretin (Fig. 2A). At pH 5.6 and pH 6.5, sideretin was the coumarin accumulating at highest levels in root exudates. However, in line with decreased CYP82C4 protein abundance (Fig. 1, B and C), sideretin secretion was strongly inhibited at pH 7.5 (Figure 2A). At this alkaline condition, scopoletin and fraxetin became the most prevalent coumarins in root exudates. Consequently, although the exudation rate of deglycosylated coumarins did not change substantially between pH 6.5 and pH 7.5 (Supplemental Fig. S3B), the alkaline condition triggered a clear shift away from sideretin and toward fraxetin (Fig. 2A).

The analysis of root extracts of wild-type plants revealed that scopolin concentrations were strongly increased by Fe starvation but were much less affected by the external pH (Supplemental Fig. S3C). This result contrasts with the pronounced regulation of *F6′H1* transcripts (Gautam et al. 2021; Supplemental Fig. S1) and protein accumulation by the external pH (Fig. 1, B and C). We then investigated how these pH-dependent changes are modulated by the activity of the different coumarin biosynthesis enzymes. In *s8h-1* mutant plants, in which conversion of scopolin to fraxin is disrupted (Rajniak et al. 2018; Siwinska et al. 2018; Tsai et al. 2018), scopolin levels in root extracts became more responsive to external pH (Supplemental Fig. S4A). Thus, these results suggest that scopolin produced by more abundant F6′H1 at high pH is efficiently converted to fraxin by S8H. Unlike scopolin in root extracts, exudation of its deglycosylated form scopoletin in wild-type plants was pH-responsive irrespective of whether Fe was supplied to the medium or not (Fig. 2A). Loss of PLEIOTROPIC DRUG RESISTANCE 9 (PDR9)-mediated export of coumarins had only minor effects on the pH-dependent changes in the concentration of coumarins in roots and root extracts (Supplemental Figs. S4A and S5A). In contrast to scopolin/scopoletin, both fraxin accumulation in roots and fraxetin exudation were strongly responsive to external pH in wild-type plants irrespective of Fe (Fig. 2A; Supplemental Fig. S3C). Notably, when further conversion of fraxetin to sideretin was prevented in the *cyp82C4-1* mutant, high fraxin concentration in root extracts and fraxetin in root exudates were achieved already at pH 6.5 (Figure 2B).

We also noticed a distinct effect of the external pH on the relative concentration of deglycosylated coumarins (i.e. % of total concentration of each pair) in root extracts of wild-type plants (Supplemental Fig. S3D). Unfortunately, it was not possible to estimate these ratios for the sideretin/siderin pair, because an authentic standard for siderin (sideretin-glycoside) is not available. For the pair scopoletin/scopolin, the root concentration of the deglycosylated form was always around 1.0% to 2.0% of the total (scopoletin + scopolin), irrespective of the external pH or supplied Fe (Fig. 2C). When Fe was not added to the growth medium, the proportion of fraxetin to the total of the pair fraxetin/fraxin increased from around 7% at pH 5.6 to 15% at pH 6.5 and up to 25% at pH 7.5. The presence of FeCl_3_ increased the percentage of the deglycosylated form (i.e. fraxetin) at pH 5.6 and 7.5 but not at pH 6.5 (Figure 2C). These results could suggest that scopolin deglycosylation occurs mainly during or right after its exudation, while fraxin undergoes significant deglycosylation already within roots. To assess the latter, we then used the *pdr9-2* mutant, in which fraxetin exudation is significantly decreased compared to wild type (Fig. 2B). However, only fraxin but not fraxetin concentration was higher in root extracts of *pdr9-2* plants compared to wild type (Supplemental Fig. S4). Consequently, the relative proportion of fraxetin to fraxin in root extracts of *pdr9-2* plants was significantly lower than in wild-type plants (Fig. 2C). Thus, these results did not provide evidence for fraxin deglycosylation within root cells.

Altogether, our results indicate that the external pH determines the amount of coumarins in roots and root exudates by modulating coumarin biosynthesis at multiple steps, while the shift in the composition of catecholic coumarins triggered by alkaline conditions is largely centered at CYP82C4, the last catalytic step in the biosynthesis pathway.

### Disruption of *MYB72* partially prevents the downregulation of *CYP82C4* at high pH

**Figure 3.**
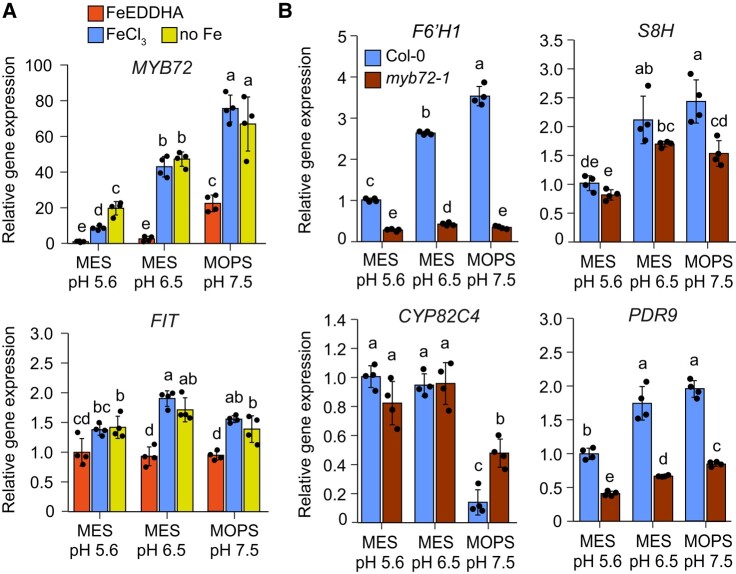
Inhibition of *CYP82C4* expression by alkaline conditions is attenuated in *myb72-1* mutant. **A)** pH-dependent regulation of *MYB72* and *FIT* transcript levels in roots of Col-0 plants grown under the indicated conditions for 4 d. Plants were precultured for 10 d on half-strength Murashige and Skoog (MS) medium with 40 *µ*M Fe-EDTA at pH 5.6 and then transferred to half-strength MS medium with 80 *µ*M FeEDDHA, 20 *µ*M FeCl_3_ (FeCl_3_) or without added Fe + 15 *µ*M ferrozine (no Fe) buffered with MES to pH 5.6 or pH 6.5, or with MOPS to pH 7.5. Relative transcript levels were normalized to *UBQ10* and *ACT2*. Bars represent means ± Sd (*n* = 4 biological replicates composed of pooled roots of 12 plants each). EDDHA, ethylenediamine-N,N′-bis(2-hydroxyphenyl)acetic acid. **B)** Relative expression of *F6′H1*, S8H, *CYP82C4*, and *PDR9* in Col-0 and *myb72-1* plants. Plants were precultured for 10 d on half-strength MS medium with 40 *µ*M Fe-EDTA at pH 5.6 and then transferred to half-strength MS medium with 20 *µ*M FeCl_3_ (FeCl_3_) buffered with MES to pH 5.6 or pH 6.5, or with MOPS to pH 7.5. Gene expression was assessed with RT-qPCR 4 d after transfer to the indicated conditions. Relative transcript levels were normalized to *UBQ10* and *ACT2*. Bars represent means ± Sd (*n* = 4 biological replicates composed of pooled roots of 12 plants each). In **A** and **B**, different letters indicate significant differences (*P* < 0.05) according to one-way ANOVA with post hoc Tukey's test (*FIT*, *S8H*, *CYP82C4*, and *PDR9*) or one-way ANOVA on ranks with post hoc Student–Newman–Keuls test (*MYB72* and *F6′H1*).

Considering that MYB72 is required for the regulation of scopoletin biosynthesis (Stringlis et al. 2018), we then investigated the role of this transcription factor in determining pH-dependent changes in coumarin composition. In line with recently reported results (Gautam et al. 2021), *MYB72* expression itself is under strong pH-dependent regulation, being continuously upregulated as the external pH is increased even when plants were supplied with the highly soluble Fe source Fe(III)-EDDHA (Fig. 3A). Much less strict pH-dependent regulation was observed for *FER-LIKE IRON DEFICIENCY INDUCED TRANSCRIPTION FACTOR* (*FIT*), which encodes the master regulator of Fe deficiency responses in *A. thaliana* (Colangelo and Guerinot 2004).

We then assessed pH-dependent regulation of coumarin-related genes in *myb72-1* plants. Compared to wild type, *myb72-1* plants were slightly larger but had lower chlorophyll concentrations at pH 5.6 (Supplemental Fig. S6). Most notably, the transcript levels of *F6′H1* and *PDR9* were significantly decreased in roots of *myb72-1* plants irrespective of the external pH while expression of *S8H* was decreased at pH 7.5 (Figure 3B). Most importantly, the inhibition of *CYP82C4* expression at pH 7.5 was largely attenuated in roots of *myb72-1* plants, remaining 3-fold higher than in wild type. Thus, these results suggest that MYB72 is necessary for the pH-dependent control of *CYP82C4* expression.

### External pH and buffering strength determine Fe(III) mobilization and reduction capacities of coumarins

The preferential biosynthesis and secretion of specific coumarins at different external pHs may suggest that plants recruit distinct activities according to the prevailing soil conditions. Although coumarins have been shown to mobilize and even reduce Fe(III) (Mladěnka et al. 2010; Schmid et al. 2014, 2014; Sisó-Terraza et al. 2016; Rajniak et al. 2018; Tsai et al. 2018), a direct comparison of the time-dependent Fe(III) mobilization and reduction capacities of scopoletin, esculetin, fraxetin, and sideretin at different pHs is lacking. Therefore, we performed in vitro assays to investigate the amount of Fe that different coumarins can mobilize and the proportion of it that becomes available in the ferrous form. Ascorbic acid and the synthetic Fe(III) chelator EDTA were used as controls for ferric Fe reduction and mobilization, respectively.

**Figure 4.**
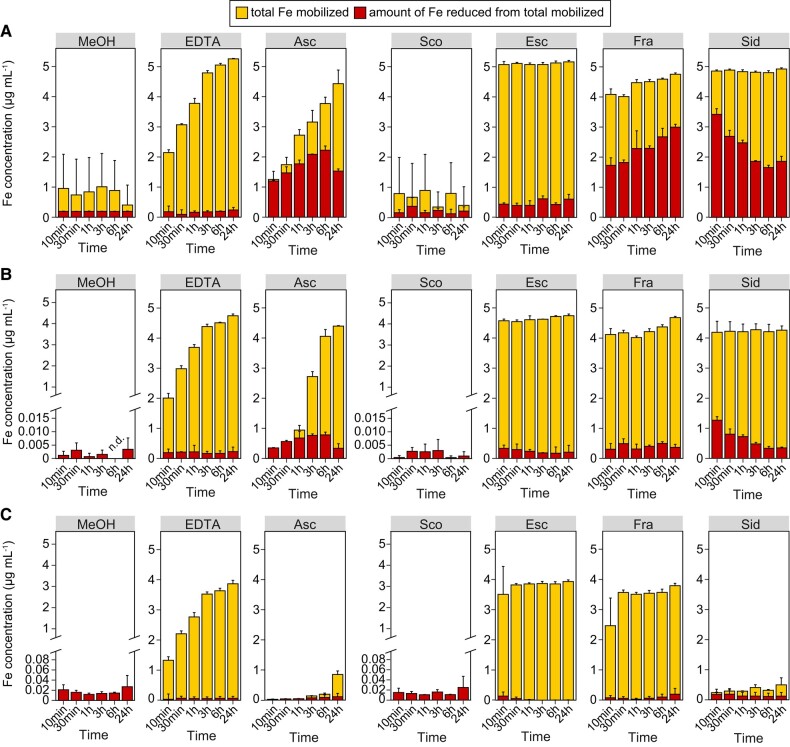
Ability of different coumarins to mobilize and reduce ferric Fe from an insoluble source at different external pH conditions. Time-dependent in vitro Fe(III) mobilization and reduction by equimolar amounts of EDTA, ascorbic acid (Asc), scopoletin (Sco), esculetin (Esc), fraxetin (Fra), and sideretin (Sid) or the solvent MeOH. Compounds were incubated in darkness with freshly precipitated Fe (supplied as FeCl_3_) at pH 5.6 **(A)**, pH 6.5 **(B)**, or pH 7.5 **(C)**. Aliquots were taken 10 min, 30 min, 1 h, 3, 6, and 24 h after starting the reaction. The amount of total mobilized Fe was determined by ICP-MS and the amount of reduced Fe was assessed spectrophotometrically on the basis of the formation of Fe(II)-ferrozine complexes. The upper end of the *y* axis represents the total amount of Fe used in the experiments. Bars represent means ± Sd (*n* = 3 reactions performed independently).

As expected, ascorbic acid showed strong ferric reduction capacity especially at low pH, while EDTA was able to mobilize but not reduce Fe(III) under all tested conditions (Fig. 4, A to C). The non-catecholic coumarin scopoletin exhibited almost no Fe(III) mobilization capacity, irrespective of the pH. In contrast, the catecholic coumarins esculetin, fraxetin, and sideretin showed distinctive capacities to mobilize and reduce Fe(III) in a highly pH-dependent manner (Fig. 4, A to C). At pH 5.6 and 6.5, esculetin, fraxetin, and sideretin were able to quickly solubilize almost all precipitated Fe (Fig. 4, A and B). However, esculetin barely reduced any of the mobilized Fe(III), while both fraxetin and sideretin reduced Fe(III) but following different kinetics. Electrochemical characterization of coumarins with cyclic voltammetry revealed that, at acidic pH, the best reductant (i.e. easiest molecule to become oxidized) was sideretin, followed by fraxetin (Supplemental Fig. S7). The estimated midpoint potentials under these conditions were 20 mV (sideretin), 284 mV (fraxetin), 395 mV (esculetin), and 697 mV (scopoletin). However, although sideretin reduced Fe(III) faster than fraxetin at pH 5.6, the amount of ferrous Fe in sideretin-containing solutions decreased over time (Fig. 4A), suggesting that putatively formed Fe(II)-sideretin complexes possess low stability allowing for reoxidation of ferrous Fe.

When the pH of the reaction solutions was increased, ferric reduction by fraxetin and sideretin was strongly inhibited (Fig. 4B). Strikingly, at alkaline pH, Fe(III) mobilization by sideretin was almost negligible (Fig. 4C). At this pH, esculetin and fraxetin were still able to mobilize up to 80% of all precipitated Fe but both coumarins lost their ability to reduce Fe(III). Fraxetin retained relatively high ferric reduction capacity at pH 6.5 and 7.5 when bicarbonate, a common pH buffer especially in calcareous soils, was used at low buffering strength (Supplemental Fig. S8). However, even the supply of higher bicarbonate concentrations did not decrease fraxetin-mediated Fe(III) mobilization while only a small fraction of the mobilized Fe was in the ferrous form (Supplemental Fig. S8). These results suggest that elevated pH and high bicarbonate concentrations inhibit coumarin-mediated ferric reduction activity or favor Fe(II) reoxidation.

### Fraxetin and sideretin can bypass FRO2-mediated enzymatic ferric reduction

In order to determine whether coumarins can facilitate ferric reduction in planta, we investigated which coumarins can alleviate Fe-deficiency symptoms of *fro2* plants under different pHs. To avoid negative effects of high pH on the availability of other nutrients, the solid agar medium contained sufficient amounts of all macro- and micronutrients while Fe was supplied as poorly soluble FeCl_3_. After 6 d of cultivation, shoot Fe concentrations of plants grown under pH 6.5 and 7.5 were significantly lower than those grown on pH 5.6 and reached critical deficiency levels (Supplemental Fig. S9). The shoot concentrations of other nutrients responded differently, with non-Fe metals that can be taken up by Fe deficiency-induced IRT1 reaching significantly higher concentrations at pH 6.5.

**Figure 5.**
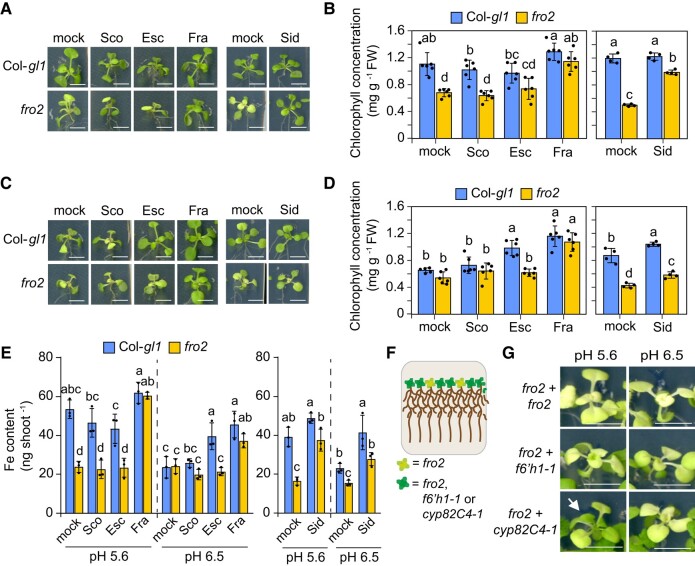
Mitigation of Fe deficiency in *fro2* by different coumarins. **A to E)** Plant appearance **(A and C)**, shoot chlorophyll concentration **(B and D)**, and shoot Fe content **(E)** of wild-type (Col-*gl1*) and *fro2* plants after 6 d of cultivation in solid agar medium supplied with different coumarins. Ten-day-old seedlings precultured on half-strength MS medium with 40 *µ*M Fe-EDTA at pH 5.6 were transferred to half-strength MS medium with 20 *µ*M FeCl_3_ buffered with MES to pH 5.6 **(A and B)** or pH 6.5 **(C and D)**. The medium was supplied with scopoletin (Sco), esculetin (Esc), fraxetin (Fra), sideretin (Sid), or only the solvent (mock). Scale bars, 5 mm. In **B and D**, bars represent means ± Sd (*n* = 4 to 6 biological replicates composed of 4 pooled shoots each). In **E**, bars represent means ± Sd (*n* = 3 biological replicates composed of 4 shoots each). In **B**, **D**, and **E**, different letters within each pH condition indicate significant differences (*P* < 0.05) according to one-way ANOVA with post hoc Tukey's test. FW, fresh weight. **F and G)** Co-cultivation of *fro2* with *cyp82C4*-1 alleviates leaf chlorosis at low pH. Schematic representation of the experiment **(F)** and shoot appearance **(G)** of *fro2* plants co-cultivated either with itself or with *f6′h1-1* or *cyp82C4-1*. Plant were precultured on half-strength MS medium with 40 *µ*M Fe-EDTA at pH 5.6 for 10 d and then transferred to half-strength MS with 20 *µ*M FeCl_3_ buffered with MES to pH 5.6 or pH 6.5 for another 11 d. Arrow indicates re-greening of young leaves of *fro2*. Scale bars, 0.5 cm.

To test chemical complementation of *fro2* by coumarins, we then supplied scopoletin, esculetin, and fraxetin at 50 *µ*M final concentration. Over the course of several days, degradation and oxidation of sideretin are two times faster than fraxetin (Rajniak et al. 2018), thus we supplied sideretin at a final concentration of 100 *µ*M to partially compensate for its loss over the experimental period. Furthermore, since sideretin has almost no detectable Fe(III) mobilization activity and is potentially destabilized at alkaline pH (Fig. 4), *fro2* complementation with sideretin was not attempted at pH 7.5. As expected from the poor Fe(III) mobilization and reduction activities in vitro, scopoletin supply was neither able to prevent Fe deficiency nor increase Fe concentration and contents of *fro2* plants irrespective of the pH conditions (Fig. 5, A to G; Supplemental Fig. S10, A and B). At pH 6.5, esculetin was only able to efficiently alleviate Fe-deficiency symptoms and increase shoot Fe content in wild-type but not in *fro2* plants, suggesting that esculetin can improve Fe acquisition only in the presence of a functional FRO2. In contrast, exogenously supplied fraxetin maintained shoot growth, prevented chlorosis, and significantly increased shoot Fe concentration and content of *fro2* plants at both pH 5.6 and pH 6.5 (Figure 5, A to D and G; Supplemental Fig. S10, A and B). At alkaline pH conditions, fraxetin lost its ability to prevent Fe deficiency of *fro2* plants but was still able to increase chlorophyll levels of wild-type plants, although not significantly increasing shoot Fe contents (Supplemental Fig. S10C to E). Sideretin, in turn, almost fully rescued the chlorosis and Fe content and concentration of *fro2* plants at pH 5.6 but was less effective at pH 6.5 (Figure 5, A to D; Supplemental Fig. S10, A and B).

To further assess the potential nonenzymatic Fe(III) reduction by coumarins without relying on chemical complementation, we performed a co-cultivation experiment (Fig. 5, F and G), in which *fro2* plants were grown next to *cyp82C4-1*, a mutant that releases approximately 130 fold more fraxetin than the wild type (Supplemental Fig. S5). We observed that co-cultivation with *cyp82C4-1* was able to significantly regreen *fro2* plants at pH 5.6 (Figure 5G; Supplemental Fig. S11). At pH 6.5, *cyp82C4-1* plants could only partially restore root growth of *fro2* but not shoot chlorophyll levels (Supplemental Fig. S11), suggesting that the amount of fraxetin exuded by *cyp82C4-1* plants was insufficient to reduce enough Fe(III) to meet *fro2*'s whole-plant demand for Fe. Altogether, these findings demonstrate that fraxetin and sideretin can bypass enzymatic ferric reduction by FRO2, with comparable efficiencies at acidic pH and with fraxetin exhibiting superior performance at near-neutral pH conditions.

### Secreted coumarin composition is altered in *fro2* plants

**Figure 6.**
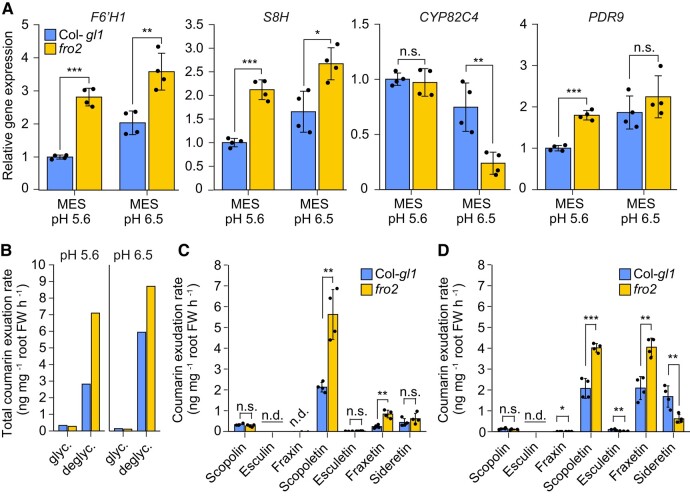
Modulation of root coumarin levels and coumarin exudation rates in *fro2* plants. **A)** Relative expression of *F6′H1*, *S8H*, *CYP82C4*, and *PDR9* in roots of wild-type (Col-*gl1*) and *fro2* plants grown for 4 d on solid half-strength MS medium without added Fe plus 15 *µ*M ferrozine, and buffered with MES to pH 5.6 or pH 6.5. Relative transcript levels were normalized to *UBQ10* and *ACT2*. Bars represent means ± Sd (*n* = 4 biological replicates composed of pooled roots of 12 plants each). **B to D)** Total exudation rate of glycosylated (glyc.) and deglycosylated (deglyc.) coumarins **(B)**, and exudation rates of individual coumarins at pH 5.6 **(C)** and pH 6.5 **(D)**. Ten-day-old wild-type (Col-*gl1*) and *fro2* seedlings precultured on half-strength strength MS medium with 40 *µ*M Fe-EDTA at pH 5.6 were transferred to half-strength MS medium without added Fe plus 15 *µ*M ferrozine, and buffered with MES to pH 5.6 or pH 6.5 for additional 4 d. Root exudates were collected for 6 h on water adjusted to the respective pH with MES. Bars represent means ± Sd (*n* = 4 biological replicates composed of pooled exudates from 140 plants each). n.d., not detected; FW, fresh weight. In **A**, **C**, and **D**, significant differences (*P* < 0.05) are indicated as asterisks according to Student's *t*-test (*P < 0.05, **P < 0.01, ***P < 0.001). n.s., not significant (*P* > 0.05).

Next, we assessed whether coumarin exudation is altered when FRO2 activity is impaired. Even when grown on no added Fe, *fro2* plants were more chlorotic and had lower Fe concentrations than the respective wild type (Supplemental Fig. S12). Consequently, the expression of *F6′H1* and *S8H* was significantly upregulated in *fro2* roots at pH 5.6 and pH 6.5, and *PDR9* was significantly induced at pH 5.6 (Fig. 6A). However, *CYP82C4* transcript levels responded differently, remaining unchanged or being repressed in *fro2* roots at pH 5.6 and 6.5, respectively. The more severe Fe-deficient status of *fro2* plants also resulted in higher exudation rates of deglycosylated coumarins in *fro2* plants compared to wild type under either pH (Fig. 6B). Notably, the downregulation of *CYP82C4* already at pH 6.5 promoted a shift in coumarin composition, as *fro2* plants released less sideretin and more fraxetin and scopoletin when grown at this pH compared to wild-type plants (Fig. 6, C and D). Thus, these results show that the absence of FRO2 favors fraxetin exudation already at near-neutral pHs.

### F6′H1-dependent coumarins play additive functions with FRO2 in ferric Fe reduction

**Figure 7.**
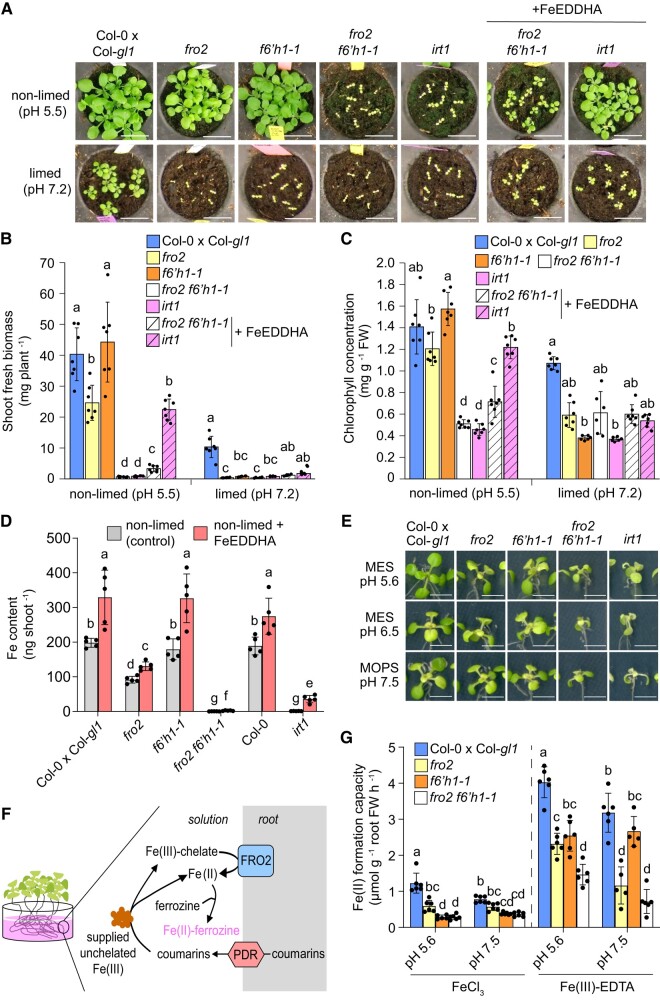
Simultaneous disruption of *FRO2* and *F6′H1* results in severe Fe deficiency and decreased Fe accumulation. **A to C)** Phenotypic analysis of *fro2 f6′h1-1* double mutant plants. Appearance **(A)**, shoot fresh biomass **(B)**, and leaf chlorophyll concentration **(C)** of wild-type (Col-0 x Col-*gl1*), *fro2*, *f6′h1-1*, *fro2 f6′h1-1* double mutant, and *irt1-1* plants grown for 19 d on non-limed substrate or on substrate limed with 20 g calcium carbonate and 12 g bicarbonate per kg substrate limed substrate. The pH values measured at the day of sowing are indicated. One set of *fro2 f6′h1-1* and *irt1-1* plants was supplemented with Fe(III)-EDDHA 3 times per week (soil application). Bars represent means ± Sd (*n* = 6 to 7 biological replicates composed of 4 to 8 pooled shoots each) and different letters within each condition represent significant differences (*P* < 0.05) according to one-way ANOVA with post hoc Tukey's test (non-limed) or one-way ANOVA on ranks with post hoc Dunn's test (limed). FW, fresh weight. Scale bars, 2 cm. **D)** Comparison of shoot Fe content of the indicated genotypes after 19 d of growth on non-limed substrate (pH ∼5.5) supplemented (FeEDDHA) or not (control) with the synthetic Fe-chelate Fe(III)-EDDHA. Bars represent means ± Sd (*n* = 4 to 5 biological replicates composed of 4 to 8 pooled shoots each) and different letters represent significant differences (*P* < 0.05) according to one-way ANOVA on ranks with post hoc Student–Newman–Keuls test. EDDHA, ethylenediamine-N,N′-bis(2-hydroxyphenyl)acetic acid. **E)** Appearance of wild-type (Col-0 x Col-*gl1*), *fro2*, *f6′h1-1*, *fro2 f6′h1-1* double mutant, and *irt1-1* plants after 6 d of cultivation under different low Fe availability conditions on agar plates. Ten-day-old seedlings precultured on half-strength strength MS medium with 40 *µ*M Fe-EDTA at pH 5.6 were transferred to half-strength MS with 20 *µ*M FeCl_3_ buffered with either MES to pH 5.6 or pH 6.5, or MOPS to pH 7.5. Quantifications of shoot biomass, chlorophyll concentration, and Fe concentration and content are presented in Supplemental Fig. S14. Scale bars, 0.5 cm. **F to G)** Schematics representing the procedure used to determine the combined Fe(II) formation capacity by membrane-bound ferric reductases and chemical reductants **(F)** and the recorded Fe(II) formation capacities of the indicated genotypes when ferric Fe provided as non-chelated (FeCl_3_) or chelated (Fe(III)-EDTA) form at slightly acidic or alkaline conditions **(G)**. For the assay, roots of intact Fe-deficient plants were placed in solutions containing the indicated Fe sources and buffered with MES (pH 5.6) or MOPS (pH 7.5) for 6 h. Bars represent means ± Sd (*n* = 5 to 6 independent replicates with 6 plants each) and different letters for each Fe source represent significant differences (*P* < 0.05) according to one-way ANOVA with post hoc Tukey's test.

Under acidic soil conditions, growth and shoot Fe concentrations of *fro2* plants are only partially inhibited and still higher when compared to the Fe(II) uptake-deficient mutant *irt1* (Supplemental Fig. S13, A and B), indicating the contribution of a FRO2-independent Fe(III) reduction mechanism. In order to directly demonstrate the functional interplay between enzymatic and nonenzymatic ferric Fe reduction in Fe acquisition, we generated a *fro2 f6′h1-1* double mutant. Interestingly, *fro2 f6′h1-1* plants exhibited very severe growth inhibition and low chlorophyll concentrations similar to the *irt1* mutant already when grown on non-limed substrate with acidic pH (Fig. 7, A to C). On limed soil with alkaline pH, single mutants were also severely Fe deficient and only wild-type plants were still able to produce approximately 25% of the biomass compared to non-limed conditions. The supply of ample amounts of the highly soluble, synthetic Fe(III)-chelate FeEDDHA to the substrate at regular intervals could largely restore the shoot biomass and chlorophyll concentration of *irt1* plants under non-limed conditions but not or much less of *fro2 f6′h1-1* plants (Fig. 7, A to C). In limed soil, Fe(III)-EDDHA supply failed to substantially improve the growth of *fro2 f6′h1-1* or *irt1* (Fig. 7, A and B).

To estimate the contribution of coumarins in Fe acquisition under slightly acidic pH, we determined shoot Fe contents in plants supplemented or not with FeEDDHA. While the absence of FRO2-mediated Fe(III) reduction in the single *fro2* mutant decreased the Fe content, the mutant still accumulated approximately 45% of the content detected in the corresponding wild type and approximately 70 times more than *irt1* plants (Fig. 7D). When both *FRO2* and *F6′H1* were lacking, shoot Fe content diminished by >95% and was similar to *irt1* plants, indicating that Fe acquisition was almost completely abolished in *fro2 f6′h1-1*. The supplementation of ample amounts of FeEDDHA significantly increased the shoot biomass and Fe content in *irt1* plants by approximately 11- and 29-fold, respectively, while the increases were limited to approximately 7.8- and 6.2-fold, respectively, in *fro2 f6′h1-1* plants (Fig. 7D; Supplemental Fig. S13C). Furthermore, FeEDDHA supplementation was able to significantly increase shoot Fe concentration only in *irt1* but not in *fro2 f6′h1-1* plants (Supplemental Fig. S13D). Together, these results indicate that even IRT1-independent Fe import into roots relies on Fe(III) reduction by at least 1 of the 2 reduction mechanisms.

Since coumarins can alter the composition of microbial communities (Stringlis et al. 2018; Voges et al. 2019; Harbort et al. 2020), potentially attracting commensals with high ferric reduction capacity, we assessed *fro2 f6′h1-1* phenotypes under axenic conditions in the presence of a non-chelated Fe source at different external pHs. Under these conditions, *fro2 f6′h1-1* plants also exhibited poor growth, low chlorophyll levels, and decreased shoot Fe content (Fig. 7E; Supplemental Fig. S14).

To better define under which pH conditions the single *fro2* and *f6′h1-1* mutants start displaying more severe symptoms of Fe deficiency, we prepared the soil substrate with different concentrations of calcium carbonate and bicarbonate resulting in gradual increases of soil pH. Only under acidic conditions *fro2* plants could sustain growth and maintain relatively high chlorophyll concentrations, while becoming severely Fe deficient already at near-neutral pH (Supplemental Fig. S15). In turn, the growth of *f6′h1-1* plants was only strongly impaired when the pH was neutral or alkaline, while the *fro2 f6′h1-1* double mutant had severe symptoms of Fe deficiency at all tested pH conditions. Thus, these results indicated that F6′H1-dependent coumarins complement FRO2-driven ferric reduction at acidic conditions.

Next, we assessed the root ferric reduction activity of plants devoid of *FRO2*, coumarins, or both. To increase the chance of detecting coumarin-mediated Fe(III) reduction, we adapted the method and incubated plants in the reaction solution containing poorly available FeCl_3_ for 6 h to enable substantial accumulation of root-released coumarins in the reaction solution (Fig. 7F). In the presence of this non-chelated Fe form, the capacity of *fro2* plants to form Fe(II) decreased by approximately 50% at pH 5.6% and 30% at pH 7.5 (Figure 7G). However, the ferric reduction capacity of *fro2* remained significantly higher than that of *f6′h1-1* or *fro2 f6′h1-1* plants, which exhibited similar capacities. To circumvent the need of coumarins to solubilize otherwise precipitated Fe, we repeated the experiment but supplied ferric Fe as chelated Fe(III)-EDTA. At acidic pH, *fro2 f6′h1-1* plants exhibited significantly lower ferric reduction capacity than *fro2* plants but the difference between these 2 mutants disappeared at pH 7.5 (Figure 7G).

Thus, our results indicate that FRO2 and coumarins together constitute the overall Fe(III) reduction capacity required for root Fe uptake in *A. thaliana*. At acidic pH, FRO2- and coumarin-dependent ferric Fe reduction are complementary while at alkaline conditions, reduction is largely dominated by FRO2.

### Nonenzymatic Fe(III) reduction is mediated by catecholic coumarins with a dominant role for sideretin at acidic conditions

**Figure 8.**
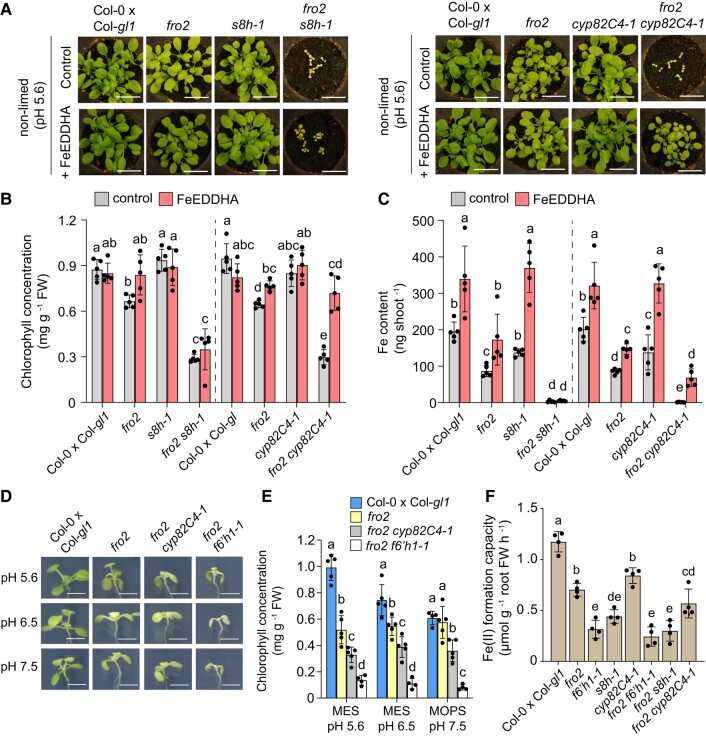
Critical role of nonenzymatic Fe(III) reduction by catecholic coumarins with a dominant role for sideretin at acidic conditions. **A to C)** Characterization of *fro2 s8h-1* and *fro2 cyp82C4-1* double mutants on slightly acidic soil substrate. Plant appearance **(A)**, leaf chlorophyll concentration **(B)**, and shoot Fe content **(C)** of indicated genotypes after 19 d of growth on non-limed substrate (pH ∼5.6) supplemented or not 3 times per week with the synthetic Fe-chelate Fe(III)-EDDHA. Bars represent means ± Sd (*n* = 5 biological replicates consisting of 5 pooled shoots each). Different letters within each genotype group represent significant differences (*P* < 0.05) according to one-way ANOVA with post hoc Tukey's test **(B)** or one-way ANOVA on ranks with post hoc Student–Newman–Keuls test **(C)**. Scale bars, 2 cm. FW, fresh weight; EDDHA, ethylenediamine-N,N′-bis(2-hydroxyphenyl)acetic acid. **D and E)***fro2 cyp82C4-1* plants are more tolerant than *fro2 f6′h1-1* to elevated external pHs. Plant appearance **(D)** and chlorophyll concentration **(E)** of the indicated genotypes after 6 d of cultivation under different external pH conditions. Ten-day-old seedlings precultured on half-strength strength MS medium with 40 *µ*M Fe-EDTA at pH 5.6 were transferred to half-strength MS with 20 *µ*M FeCl_3_ buffered with either MES to pH 5.6 or pH 6.5, or MOPS to pH 7.5. Bars represent means ± Sd (*n* = 4 to 5 biological replicates composed of 4 shoots each) and different letters within each pH condition indicate significant differences (*P* < 0.05) according to one-way ANOVA with post hoc Tukey's test. Scale bars, 0.5 cm. FW, fresh weight. **F)** Fe(II) formation capacity of indicated genotypes after 4 d of growth on Fe-depleted medium (no added Fe + 15 *µ*M ferrozine, pH 6.5 buffered with MES). For the assay, roots of intact plants were placed in a solution containing FeCl_3_ buffered at pH 6.5 with MES for 6 h. Bars represent means ± Sd (*n* = 4 biological replicates with 6 plants each) and different letters represent significant differences (*P* < 0.05) according to one-way ANOVA with post hoc Tukey's test. FW, fresh weight.

Since *s8h* mutants exude high levels of scopoletin but virtually none of the catecholic coumarins fraxetin and sideretin (Rajniak et al. 2018; Supplemental Fig. S5), we also generated a *fro2 s8h-1* double mutant to investigate the relevance of the non-catecholic coumarin scopoletin in Fe(III) reduction in planta. Since the importance of ferric reduction by coumarins was largely restricted to acidic conditions, we concentrated the phenotypic analysis to non-limed soil with pH ∼5.6. Under this condition, the *fro2 s8h-1* double mutant exhibited substantially weaker growth and much lower chlorophyll concentration and shoot Fe concentration and content compared to wild-type and single mutant plants (Fig. 8, A to C; Supplemental Fig. S16). Similar to *fro2 f6′h1-1*, growth, chlorophyll levels, and Fe accumulation of *fro2 s8h-1* plants could be barely improved by soil fertilization with Fe(III)-EDDHA. These results further reinforced that scopoletin has limited contribution for nonenzymatic ferric Fe reduction and highlighted a major role for the catecholic moiety in coumarins in this process.

Our in vitro assays demonstrated that both fraxetin and sideretin exhibit comparable Fe(III) mobilization and reduction capacities at low pH. Thus, it remained elusive why the exudation of sideretin is preferred over fraxetin under acidic conditions, especially considering that Fe acquisition and plant growth are not significantly impaired in sideretin-deficient *cyp82C4-1* mutant plants (Rajniak et al. 2018). Taking advantage of the high fraxetin and nearly lacking sideretin exudation of *cyp82C4* mutant plants (Supplemental Fig. S5), we generated a *fro2 cyp82C4-1* double mutant to investigate whether the comparatively small differences in Fe(III) reduction kinetics between sideretin and fraxetin have any physiological relevance for Fe acquisition. Although the fraxetin exudation rate of the *fro2 cyp82C4-1* double mutant was around 80% less than that of the *cyp82C4-1* single mutant, *fro2 cyp82C4-1* plants exuded approximately 20 times more fraxetin and 98% less sideretin compared to wild-type and *fro2* plants (Supplemental Fig. S17). However, despite the high fraxetin exudation, *fro2 cyp82C4-1* plants exhibited poor growth, severe chlorosis, and very low shoot Fe concentration and contents (Fig. 8, A to C; Supplemental Fig. S16).

Similar to *fro2 f6′h1-1*, the Fe-deficient phenotypes of *fro2 s8h-1* and *fro2 cyp82C4-1* double mutants were severe already at acidic pH and not further exacerbated at higher pHs (Supplemental Fig. S15). However, unlike *fro2 f6′h1-1* and *fro2 s8h-1*, chlorophyll levels and shoot Fe concentration of the *fro2 cyp82C4-1* double mutant were significantly improved by supplying Fe(III)-EDDHA via soil fertilization (Fig. 8, A and B; Supplemental Fig. S16). Within 19 d of growth, the improved Fe status of FeEDDHA-supplied *fro2 cyp82C4-1* double mutant plants allowed these plants to reach 27% and 33% of the shoot biomass and Fe content, respectively, of the corresponding wild type (Fig. 8C). Thus, these results suggest that fraxetin exuded by *fro2 cyp82C4-1* plants was insufficient to mobilize and reduce sufficient soil Fe(III) but was relatively efficient in reducing ferric Fe from a chelated source.

To estimate the contribution of fraxetin in Fe acquisition at elevated pH conditions, we grew *fro2 cyp82C4-1* and *fro2 f6′h1-1* in solid agar medium containing poorly available Fe and adjusted to different pH values. At pH 5.6, *fro2 cyp82C4-1* and *fro2 f6′h1-1* double mutants showed similarly impaired growth and only slightly higher chlorophyll levels (Fig. 8, D and E; Supplemental Fig. S18). However, growth and chlorophyll levels of the *fro2 cyp82C4-1* double mutant slightly improved when the external pH was raised to 6.5 or 7.5. We then assessed the ferric Fe reduction capacity of all double mutants at pH 6.5. Under this condition, Fe(II) formation of *fro2 cyp82C4-1* plants (secrete scopoletin and high fraxetin) was significantly higher than *fro2 f6′h1* (no coumarins) and *fro2 s8h-1* (only scopoletin), although still significantly lower than *fro2* single mutant (all coumarins) (Fig. 8F).

Taken together, these results demonstrate that fraxetin-mediated Fe(III) reduction is increasingly important at higher external pH conditions, while sideretin has a critical role at acidic pH, which cannot be replaced by fraxetin except if large amounts of chelated Fe(III) are present in the growth medium.

## Discussion

Exudation of coumarins has been established as a key component of the Fe acquisition machinery of *A. thaliana*. Previous phenotypic characterization of mutants impaired in coumarin biosynthesis or export indicated that coumarins are critical for survival on alkaline substrates or even on acidic to slightly acidic pH when Fe is present predominantly in a poorly soluble form (Rodriguez-Celma et al. 2013; Fourcroy et al. 2014; Schmid et al. 2014; Schmidt et al. 2014; Rajniak et al. 2018; Tsai et al. 2018). Thereby, the critical role of coumarins has mainly been defined by their ability to chelate Fe(III) and still largely focused on fraxetin. However, it has remained unclear why Fe-deficient *A. thaliana* plants exude different types of coumarins, especially considering that some of them, such as fraxetin and sideretin, differ only little at the structural level. Although small, these differences are likely relevant as sideretin release is preferred at acidic and fraxetin at alkaline pH conditions (Fig. 2; Sisó-Terraza et al. 2016; Rajniak et al. 2018; Gautam et al. 2021). In this study, we (i) provide insights into how the environmental pH affects the abundance of coumarin biosynthesis enzymes to adjust the amount and composition of different coumarins in root exudates, (ii) demonstrate a major mechanistic function of coumarins in Fe(III) reduction besides their ability in Fe chelation, and (iii) establish the dominant physiological roles of sideretin and fraxetin at different pH conditions.

### Environmental pH modulates coumarin composition of root exudates at multiple levels

Besides being upregulated in response to Fe deficiency, the transcript and protein levels of *F6′H1*, *S8H*, and *CYP82C4* are additionally controlled by the environmental pH (Supplemental Fig. S1; Gautam et al. 2021). Based on the localization of the three major coumarin biosynthesis enzymes (Fig. 1, B and C), coumarins are mainly synthesized in the cytosol of epidermal and cortical cells. The cell type-specific localization of F6′H1 and S8H partially matches the localization of scopolin and fraxin determined microscopically on the basis of the distinct emission spectra of these coumarins (Robe et al. 2021). At acidic pH conditions, the presence of all three enzymes in epidermal cells enables the efficient conversion of scopolin to fraxin and of fraxin to sideretin-glycoside (Figs. 1 and 2A). Of note, we detected relatively high levels of scopolin in root extracts irrespective of the external pH (Supplemental Fig. S3). Apart from putative differences in the catalytic activity of the three enzymes, the relatively more abundant F6′H1 in cortical cells at pH 5.6 and 6.5 may also suggest that not all scopolin undergoes further conversion in these cells. As the external pH rises, S8H abundance increases converting more scopolin into fraxin. However, our results further strengthen that the pH-dependent control of the coumarin composition of root exudates is centered on CYP82C4.

As reported recently, alkaline pH strongly inhibits *CYP82C4* already at the transcriptional level (Tsai and Schmidt 2020; Gautam et al. 2021). This strict pH-dependent regulation of *CYP82C4* results in fraxetin becoming more abundant than sideretin in root exudates of plants grown at alkaline conditions. However, still little is known about the regulation of coumarin biosynthesis genes by the environmental pH. The upregulation of coumarin biosynthesis related genes by Fe deficiency is dependent on FIT (Colangelo and Guerinot 2004; Schmid et al. 2014). Thus, a pH-responsive signal may differentially modulate FIT-dependent regulation of *F6′H1*, *S8H*, and *CYP82C4*. Here, we show that repression of *CYP82C4* by alkaline pH is strongly attenuated in roots of *myb72-1* plants (Fig. 3B), suggesting that MYB72 is required for the pH-dependent shift in coumarin composition. Since the strong downregulation of *F6′H1* in *myb72* plants results in severely compromised scopolin biosynthesis (Stringlis et al. 2018), we were not yet able to demonstrate whether the detected *CYP82C4* misregulation maintains higher sideretin release at alkaline conditions in *myb72-1* plants. So far, our results suggest that MYB72 has a direct or indirect negative impact on *CYP82C4* expression at alkaline pH while exerting a positive regulation on *F6′H1*, *S8H*, and *PDR9* irrespective of the external pH. Interestingly, *MYB72* itself is very responsive to pH with highest expression detected at alkaline pH (Fig. 3A; Gautam et al. 2021). Thus, it will be of interest to investigate whether MYB72 may have distinct effects on different steps of coumarin biosynthesis and whether it can act as a positive regulator of *F6′H1* and *S8H* and as a repressor of *CYP82C4*, specifically at alkaline conditions.

### Fe(III) mobilization and reduction capacities of different coumarins are distinctly affected by medium pH and buffering capacity

The characterization of *pdr9* mutant plants, defective in coumarin export (Fourcroy et al. 2014), demonstrated that coumarins exert their function in Fe acquisition after secretion from root cells. Once in the root apoplast or in the surrounding soil, it has been assumed that coumarins can interact with precipitated or even chelated Fe to maintain ferric Fe in solution or by forming ferrous Fe. Up to date, Fe mobilization from freshly precipitated Fe-hydroxide has only been detected for the catecholic coumarins esculetin, fraxetin, and sideretin (Fig. 4; Schmid et al. 2014; Sisó-Terraza et al. 2016; Rajniak et al. 2018). Nonetheless, significant amounts of Fe were mobilized from the Fe mineral lepidocrocite after incubation with the non-catecholic scopoletin (Baune et al. 2020). However, Fe mobilization by scopoletin is likely also mediated by a catecholic coumarin, as it was found that scopoletin undergoes demethylation at the surface of lepidocrocite thereby forming esculetin (Baune et al. 2020). The relevance of scopoletin demethylation for Fe acquisition may be limited or restricted to particular soil conditions, since *s8h* mutants, which exude large amounts of scopoletin but almost no catecholic coumarins (Supplemental Fig. S5), grow poorly in alkaline soil or in the presence of precipitated Fe at slightly acidic conditions (Rajniak et al. 2018; Tsai et al. 2018). Scopoletin can nonetheless indirectly contribute to Fe acquisition by shaping root-associated microbial communities (Stringlis et al. 2018). Esculetin, in turn, although exhibiting high Fe mobilization capacity is present at much lower concentrations in root exudates compared to the other catecholic coumarins (Fig. 2). Thus, at least in *A. thaliana*, fraxetin and sideretin play the dominant role in direct Fe mobilization.

The preferential secretion of sideretin at acidic and of fraxetin at alkaline conditions suggests that they undertake specialized functions under different pH conditions. In terms of Fe mobilization, both fraxetin and sideretin exhibit comparable capacities at acidic to near-neutral pHs (Fig. 4). To mobilize Fe, coumarins must first interact with Fe atoms of sparingly soluble (hydr)oxides or minerals. Theoretically, Fe ions can coordinate with up to three deprotonated catechol groups but the coordination mode and stability constants of the formed complexes depends on the characteristic structure of each coumarin and the external pH (Perron and Brumaghim 2009). However, it still must be determined which Fe-fraxetin and Fe-sideretin complexes are formed, especially in root exudates, and what is their predominant stoichiometry at different pHs. The severely compromised Fe mobilization capacity of sideretin at pH 7.5 (Figure 4) indicates that the physiological function of this coumarin under alkaline conditions becomes irrelevant. In contrast, fraxetin retains high Fe mobilization capacity at alkaline pH, even under strong buffering conditions.

Sideretin is quickly oxidized in the presence of oxygen, forming a quinone with poor Fe mobilization capacity (Rajniak et al. 2018). Since coumarin oxidation becomes easier as the pH increases, the high susceptibility of sideretin to oxidation could explain the lacking Fe mobilization capacity of sideretin at alkaline pH. Furthermore, alkaline conditions increase the overall susceptibility of coumarins toward opening of their lactone rings (Orlov and Piskareva 1974) and the additional hydroxyl group of sideretin compared to fraxetin could potentially facilitate this process. The higher susceptibility of sideretin to alkaline pH-induced oxidation and degradation may explain why CYP82C4 abundance and the consequent biosynthesis and release of sideretin are strongly repressed at pH 7.5. In contrast, fraxetin retains high Fe mobilization capacity at alkaline pH, even under strong buffering conditions.

Besides chelating Fe(III) or Fe(II), coumarins can also reduce ferric Fe (Fig. 4; Sisó-Terraza et al. 2016; Rajniak et al. 2018). Upon binding of a catecholate (i.e. deprotonated) coumarin with Fe(III), Fe(II) is formed and the ligand is oxidized to a semiquinone (Hider et al. 2001). At slightly acidic to near-neutral pHs, our results demonstrate that sideretin maintains a higher proportion of mobilized Fe in the ferrous form compared to fraxetin, especially within short-time scales (Fig. 4). The midpoint redox potentials of sideretin estimated at acidic (Supplemental Fig. S7) and near-neutral pHs (Rajniak et al. 2018) are lower than that of fraxetin, reinforcing that sideretin is a better reductant than fraxetin outside alkaline conditions. Although the differences between sideretin and fraxetin are not dramatic, they might be determinant in the rhizospheric environment, as quick Fe reduction by sideretin may allow plants to increase the pool of ferrous Fe near the root surface for subsequent uptake. One important finding from our in vitro assays is that while coumarin-mediated Fe mobilization was largely insensitive to pH (except for sideretin at alkaline pH), the amount of ferrous Fe was strongly decreased at near-neutral to alkaline pHs and under strong buffering conditions. At acidic conditions, the coumarin semiquinone can remain protonated, which helps to stabilize Fe(II). However, when the pH is elevated, the formation of complexes of two or three coumarins with a single Fe may slow down ferric Fe reduction. Furthermore, as catecholate ligands typically form more stable complexes with trivalent than divalent ions (Perron and Brumaghim 2009), coumarins may allow the autooxidation of complexed Fe(II) especially under elevated pH conditions.

### Sideretin and fraxetin have distinct functions in Fe mobilization at acidic and alkaline pH conditions

Our in vitro assays and chemical complementation experiments demonstrated that coumarin-mediated ferric Fe reduction is only effective at acidic pH conditions (Figs. 4 and 5, A and B). This pH-dependency may explain why *fro2* plants could not be complemented with coumarins in a previous study, as complementation was only attempted at alkaline pH (Fourcroy et al. 2016). Here, we show that the simultaneous disruption of *FRO2* and coumarin biosynthesis in a *fro2 f6′h1-1* double mutant leads to strong Fe-deficiency symptoms and severely impaired Fe accumulation (Fig. 7, A to D; Supplemental Fig. S15). Since ferric Fe reduction was more strongly compromised in the double mutant than in the *fro2* single mutant, our results demonstrate that, outside the alkaline range, Fe(III) is reduced in roots by coumarin-type reductants and membrane-bound FRO2. Ferric reduction by reductants is not exclusive to coumarins, as ascorbate-driven Fe(III) reduction from Fe-citrate-malate complexes is critical for Fe import into embryos (Grillet et al. 2014). However, it remains to be determined whether coumarins may also reduce Fe(III) present in other soil-borne complexes besides the ferric Fe they mobilize by themselves. Coumarin-mediated Fe(III) reduction may become even more relevant under conditions that inhibit FRO2 activity even at low pH, such as excess copper (Barton et al. 2000; Waters and Armbrust 2013), or in natural accessions with inherently lower *FRO2* expression and ferric-chelate reductase activity (Satbhai et al. 2017). Importantly, irrespective of whether ferric Fe is reduced by FRO2 or coumarins, the partial complementation of *irt1* but not of *fro2 f6′h1-1* or *fro2 s8h-1* with large amounts of chelated Fe (Figs. 7D to F and 8A to C) demonstrates that root Fe uptake by other transporters, such as NATURAL RESISTANCE-ASSOCIATED MACROPHAGE PROTEIN 1 (NRAMP1), also relies on the availability of reduced Fe.

Although it is difficult to directly quantify the relative contribution of FRO2- and coumarin-mediated Fe(III) reduction in wild-type plants, the almost indistinguishable phenotypes of the *fro2 f6′h1-1* double mutant and *irt1* indicate that virtually no ferric Fe reduction capacity remains in roots when FRO2 activity and coumarin biosynthesis are simultaneously disrupted. By subtracting the Fe(II) formed by *fro2* and *fro2 f6′h1-1* plants supplied with Fe(III)-EDTA from the total amount formed by wild-type plants (Fig. 7E), the estimated relative contribution of FRO2- and coumarin-mediated ferric reduction was ∼42% and 20%, respectively, at pH 5.6 and ∼64% and 14% at pH 7.5. Obviously, these numbers represent rough estimations, as (i) they do not consider possible chelator exchange aspects, (ii) exudation rates of coumarins are higher in *fro2* than in wild-type plants (Fig. 6), and (iii) our ferric reduction assays do not simulate relevant rhizosphere conditions, including microbial activities, coumarin gradients and H^+^-ATPase-formed acidic microenvironments.

Whereas at the structural level fraxetin and sideretin are very similar, the higher secretion of sideretin at low pH suggests a more critical role of this coumarin under acidic conditions. The phenotypic analysis of a *fro2 cyp82C4-1* mutant allowed us to demonstrate that sideretin is indeed superior over fraxetin in driving ferric Fe reduction at acidic pH (Fig. 8, A to C). One possibility to explain this apparent advantage is that the faster Fe(III) reduction kinetics of sideretin allows it to more efficiently access Fe pools in the apoplast or in the rhizosphere soil, increasing the chance that the formed ferrous Fe is taken up. In turn, fraxetin must be present at high concentrations in root exudates or have access to large amounts of Fe to partially complement the lack of sideretin. Furthermore, the comparatively lower amount of ferrous Fe formed by fraxetin within short-time spans indicates that reduction of ferric Fe mobilized by fraxetin is more dependent on FRO2 activity.

In fact, our results demonstrate that fraxetin becomes more relevant for Fe acquisition at alkaline conditions. However, under these conditions, instead of reducing Fe(III), the main function of fraxetin is to provide soluble Fe(III)-chelates for FRO2-mediated reduction. This conclusion is supported by the fact that fraxetin cannot complement *fro2* at alkaline pH and because growth on limed soil substrate can only be sustained when both *FRO2* and coumarin biosynthesis are not disrupted (Fig. 7, A to C; Supplemental Fig. S15). Although enzymatic ferric-chelate reductase activity is also negatively affected by alkaline pH and high bicarbonate concentrations (Romera et al. 1992; Susin et al. 1996; Lucena et al. 2007), the conditions in which the membrane-bound FRO2 can operate are still more favorable than those in the apoplast or in the rhizospheric soil, where released coumarins accumulate. This is because FRO2 is present in the plasma membrane as a protein complex with AHA2 and IRT1 (Martín-Barranco et al. 2020), suggesting that the protons extruded by AHA2 can sustain a more acidic microenvironment near FRO2.

Thus, under alkaline conditions, we propose that fraxetin mobilizes and stabilizes ferric Fe which can then serve as substrate for enzymatic reduction via FRO2 situated within the acidic milieu formed by AHA2 activity. Our results also indicate that fraxetin can likely exert this activity even in the presence of high concentrations of bicarbonate (Supplemental Fig. S8). High Fe(III) mobilization could explain why *A. thaliana* accessions with higher fraxetin exudation capacity are more tolerant to alkaline pHs and high soil carbonate concentrations (Tsai et al. 2018; Terés et al. 2019). A putative uptake of Fe-fraxetin complexes seems less likely. While fraxetin concentrations increased in root extracts at pH 7.5 (Fig. 2C), potentially indicating re-uptake by roots, *irt1* plants exhibited severe Fe deficiency and extremely low Fe contents irrespective of the external pH (Fig. 7, A to D; Supplemental Fig. S14). Furthermore, fraxetin became unable to complement *fro2* plants at pH 7.5 (Fig. 5, E to I), when the stability of fraxetin-Fe complexes should be high. Thus, uptake of intact fraxetin-Fe complexes, if existing, makes an almost negligible contribution to Fe uptake in roots.

## Materials and methods

### Plant materials

In this study, the *A. thaliana* EMS mutant *fro2* (*frd1-1*) (Yi and Guerinot 1996) and the corresponding wild-type Columbia *glabra-1* (Col-*gl1*) were used. Furthermore, the following homozygous T-DNA-insertion lines from the SALK, SM, or GABI collection (all in Col-0 background) were used: *f6′h1-1* (SALK_132418C; Schmid et al. 2014), *s8h-1* (SM_3_27151; Rajniak et al. 2018), *s8h-2* (SM_3_23443; Rajniak et al. 2018), *cyp82C4-1* (SALK_001585; Rajniak et al. 2018), *irt1* (Varotto et al. 2002), *pdr9-2* (SALK_050885; Fourcroy et al. 2014), and *myb72-1* (SAIL_713G10; Zamioudis et al. 2014). To generate the *fro2 f6′h1-1* double mutant, *fro2* was crossed with *f6′h1-1*, and homozygous *fro2*, *f6′h1-1*, *fro2 f6′h1-1*, and as well wild-type (designated Col-0 x Col-*gl1*) plants selected from the F2 population with PCR with primers listed in Supplemental Table S1 and by confirming the expected point mutation in the *FRO2* locus (Supplemental Fig. S19). A similar strategy was used to generate *fro2 s8h-1* and *fro2 cyp82C4-1* double mutants. All homozygous mutant lines were selfed and seeds from F3 generations were used in all experiments.

### Growth conditions

In agar plate experiments, *A. thaliana* seeds were surface sterilized with a solution containing 70% (v/v) EtOH and 0.05% (v/v) Triton-X-100 (Roth). Sterilized seeds were precultured on sterile agar plates containing one-half strength Murashige and Skoog (MS) medium with 40 *µ*M NaFeEDTA, supplemented with 0.5% (w/v) sucrose and 2.5 mM MES pH 5.6, and solidified with 1% (w/v) Difco agar (Becton Dickinson). Ten days after germination, seedlings were transferred to fresh, solid one-half strength MS media supplemented with either freshly prepared 20 *µ*M FeCl_3_ (Roth) or no added Fe plus 15 *µ*M ferrozine (Serva), and pH buffered to 5.6 or 6.5 with 2.5 mM MES, or to 7.5 with 1.25 mM MOPS. Buffers were prepared as 500 mM stocks and the pH adjusted to desired value with 1 mM NaOH. Plants were assessed after 4 or 6 d of cultivation on treatments depending on the experiment (see figure legends). Plates were kept vertically inside a growth cabinet under a 22 °C/18 °C and 10/14 h light/dark regime with the light intensity of fluorescent bulbs adjusted to 120 *µ*mol photons m^−2^ s^−1^. In the co-cultivation experiment, each *fro2* plant was surrounded by 4 plants of the co-cultivating genotype. The plates were kept vertically in the growth cabinet and *fro2* plants were harvested after 11 d.

In chemical complementation experiments, coumarins were dissolved in methanol (MeOH). Sideretin was freshly reduced with hydrogen gas and palladium following the procedure described previously (Rajniak et al. 2018). Coumarins were added to autoclaved medium after cooling down. Equal amounts of MeOH were used as solvent mock controls.

Phenotyping of plants on peat-based substrate was performed as described previously (Schmid et al. 2014). In brief, plants were cultivated on a peat-based substrate (Klasmann-Deilmann, Substrate 1, Germany) inside a climatized growth chamber with 22 °C/18 °C and 16/8 h light/dark regime with the light intensity of fluorescent bulbs adjusted to 120 *µ*mol photons m^−2^ s^−1^. The substrate was used without further amendments (non-limed) or limed with different concentrations of calcium carbonate and bicarbonate as indicated in the corresponding figures. One week after liming and before sowing the seeds, the pH of the substrate was determined by suspending a representative aliquot of the substrate batches in 10 mM CaCl_2_ in a 1:5 (weight:volume) proportion (Schofield and Taylor 1955). The pH values measured at the beginning of the experiments are indicated in the corresponding figures. Plants were sown in 54-pot trays with a volume of approximately 100 mL per pot (5 cm height × 2.5 cm radius). The number of plants per pot was reduced to 8 to 10 when plants were 6 d old. FeEDDHA treatment was performed by pipetting 2 mL of 0.5 g L^−1^ FeEDDHA (Duchefa Biochemie) 3 times a week directly onto soil, starting 5 d after germination. The plants were harvested 19 d after sowing.

### Cloning and plant transformation

To generate translational fusions of the proteins of interest with green fluorescent protein (GFP) in the C-terminus, we used the GreenGate modular cloning system (Lampropoulos et al. 2013). The *proF6′H1:F6′H1-GFP* construct contained a 1,166-bp fragment of the *F6′H1* open reading frame and a 2,077-bp fragment of the *F6′H1* promoter. The *proS8H:S8H-GFP* construct was created by cloning a 1,534-bp fragment of the *S8H* open reading frame and a 2,023-bp fragment of the promoter, and *proCYP82C4:CYP82C4-GFP* by cloning a 1,924-bp fragment of the *CYP82C4* open reading frame and a 2,058-bp fragment of the respective promoter. Gene specific promoter and open reading frame fragments were amplified from genomic DNA (gDNA) of *A. thaliana* accession Col-0 (CS60000). Amplification was performed using the Phusion High-Fidelity DNA Polymerase (New England Biolabs) and the primers listed in Supplemental Table S1. Promoters and open reading frame amplicons were cloned into the GreenGate entry vectors pGGA000 and pGGC000, respectively, using *Bsa*I restriction enzyme (Lampropoulos et al. 2013). Correct integration of the fragments was verified by restriction digestion reactions and the cloned sequences verified by sequencing. The individual entry modules were assembled into the GreenGate binary vector pGGZ001 including a phosphinotricin or hygromycin resistance cassette for antibiotic selection in plants. All GreenGate entry vectors, the binary vector pGGZ001 and GreenGate modules containing GFP for C-tag fusion, plant terminators, and plant resistance cassettes were purchased from Addgene (https://www.addgene.org/). The final binary vectors were introduced into the *Agrobacterium tumefaciens* strain GV3101 containing the helper plasmid pSOUP and finally transferred to *A. thaliana* (Col-0; CS60000) plants via the flower dip method (Clough and Bent 1998). One representative transgenic line for each construct was selected from 6 to 8 independent lines and used for analysis.

### Microscopy analyses

For protein localization studies, root segments of representative *proF6′H1:F6′H1-GFP*, *proS8H:S8H-GFP*, and *proCYP82C4:CYP82C4-GFP* lines were excised, stained with propidium iodide (10 *µ*g mL^−1^) for 10 min, and mounted in water. GFP- and propidium iodide-dependent fluorescence was acquired with a confocal laser-scanning microscope (LSM 780; Zeiss, Germany) equipped with a 20×/0.8 M27 objective. GFP was excited with a 488 nm Argon laser with intensity and gains always set to 2.0 and 600, respectively. Propidium iodide was excited at 561 nm with a diode-pumped solid-state laser with intensity set to 2.0 and gain variably adjusted between 550 and 650 to obtain optimal signal intensities even when root staining was less efficient. GFP- and propidium iodide-derived fluorescence emissions were detected with a 505 to 535 nm and 600 to 700 nm band-pass filters, respectively. Z-stacks were acquired using ZEN Black (Zen 2.3 SP1 FP1) while orthogonal views of Z-stacks were prepared with ZEN 2.6 (blue edition) software.

### Shoot chlorophyll analysis

Whole shoot samples were weighed and incubated for 1 to 2 d at 4 °C in N,N′-dimethylformamide (Roth). The absorbance of the extracts was measured at 647 and 664 nm (UV5Bio, Mettler Toledo, Germany) and the chlorophyll concentration was determined as described previously (Porra et al. 1989).

### Shoot mineral element analysis

For Fe and multielement analysis, whole shoot samples were dried at 65 °C and weighted into polytetrafluoroethylene tubes. Plant material was digested with concentrated HNO_3_ (67% to 69% (v/v); Bernd Kraft) and pressurized in a high-performance microwave reactor (UltraCLAVE IV, MLS GmbH). Digested samples were diluted with de-ionized water (Milli-Q Reference A+, Merck Millipore). Element analysis was carried out by a sector field inductively coupled plasma mass spectrometry (HR-ICP-MS) (ELEMENT 2, Thermo Scientific, Germany) with Software version 3.1.0.236 and the parameters indicated in Supplemental Table S2.

### Fe mobilization and reduction by coumarins

Pure coumarin standards dissolved in MeOH (final concentration of 0.4 mM) were incubated with 0.1 mM FeCl_3_ buffered with either 2.5 mM MES to pH 5.6 and 6.5, 1 mM, 2 mM, or 2.5 mM NaHCO_3_ to pH 6.5 and 7.5, or 1.25 mM MOPS to pH 7.5. Samples were incubated at room temperature in darkness on a gyratory shaker. Aliquots were taken after 10 min, 30 min, 1 h, 3, 6, and 24 h and directly filtered through Chromafil CA-45/25 (0.45 *µ*m pore) filters. NaEDTA (Merck), ascorbic acid (Sigma Aldrich), and MeOH were used as references. The Fe concentration in the filtrates was determined by (HR)-ICP-MS. The concentration of reduced Fe was determined using ferrozine. To do so, the absorbance of an aliquot of each filtrate was determined at 562 nm (UV5Bio, Mettler Toledo, Germany) before and after incubation with 0.4 mM ferrozine for 10 min in darkness at room temperature. The difference between both absorbance values was used for Fe(II) quantification against a calibration curve acquired for each individual buffer. To fully reduce Fe(III), each calibration standard was incubated with dithiothreitol for 5 h in darkness at room temperature followed by 10 min incubation with 0.4 mM ferrozine. The absorbance was determined at 562 nm and a linear regression curve calculated. Three replicates per dilution were included.

### Root ferric reduction capacity

To estimate ferrous Fe formation from membrane-bound reductases and coumarins, we made modifications in a previously published protocol (Waters et al. 2006). Roots of intact plants were incubated in a solution containing 0.2 mM CaSO_4_, 50 *µ*M Fe(III)-EDTA or FeCl_3_, and pH buffered to 5.6 or 6.5 with 2.5 mM MES, or to 7.5 with 1.25 mM MOPS. During incubation, plants were kept inside growth cabinets with the root-containing solution protected from light. To estimate the ferric reduction capacity of combined membrane-bound ferric-chelate reductases and secreted coumarins, the incubation was maintained for 6 h to allow for sufficient accumulation of root-borne coumarins in the solution. Fe(II) concentrations were determined by measuring the absorbance at 562 nm after adding 200 *µ*M ferrozine. The concentration of Fe(II)-ferrozine complexes was calculated using 28.6 mmol dm^−3^ cm^−1^ as molar extinction coefficient (Yi and Guerinot 1996).

### Collection of root exudates and sampling of roots

Root exudates were collected as described previously (Schmid et al. 2014) with small modifications. One hour after the beginning of the light phase, plants grown on different Fe and pH conditions in solid agar medium were carefully transferred to flat-bottom, 12-well plates containing 5 mL in each well to ultrapure water with pH adjusted and buffered accordingly. The well plates were placed inside trays covered with polyvinyl chloride film to prevent dehydration. The trays were kept at 22 °C, with constant illumination (120 *µ*mol photons m^−2^ s^−1^). After 6 h of collection, sampling solution was immediately frozen and stored at −80 °C until freeze drying. Plant roots were harvested and snap frozen in liquid nitrogen. Freeze-dried root exudate samples were resolved in a total volume of 10 mL 100% MeOH and vortexed vigorously. 4-Methyldaphnetin dissolved in MeOH (2 *µ*L of 100 *µ*g mL^−1^) was added as internal reference. The samples were centrifuged at 4 °C and 1,252 × *g* for 1 min, filtered through Chromafil CA-45/25 (0.45 *µ*m pore) filters, and finally concentrated to 0.5 mL using a centrifugal evaporator (Christ, Germany). Concentrated root exudate samples were stored at −80 °C until analysis. Phenolic components were extracted from frozen roots by adding 100% MeOH containing 0.4 *µ*g mL^−1^ of the internal standard 4-methyldaphnetin. Tissues were homogenized with stainless steel balls (3.0 to 3.3 mm) by vortexing vigorously. The samples were incubated overnight at 4 °C in darkness. After centrifugation at 4 °C and 16,100 × *g* for 10 min, the supernatant was collected, transferred into a new tube, and stored at −20 °C. A second extraction was performed for 1.5 to 2 h under the same conditions. The samples were centrifuged and the supernatant pooled with the first one. Prepared root extracts were stored at −20 °C until measurement.

### Coumarin quantification by UPLC-ESI-MS

Root exudate and extract samples were subjected to ultra-performance liquid chromatography mass spectrometry (UPLC-MS) analysis using a 1290 Infinity II LC system (Agilent Technologies, United States) coupled to a 6490 Triple Quad LC/MS with iFunnel technology (Agilent Technologies, United States). The Agilent UPLC system was equipped with a reversed phase Acquity UPLC HSS T3 Column (100 Å, 2.1 mm × 150 mm, 1.8 *µ*m, Waters) and an Acquity UPLC HSS T3 VanGuard Pre-column (100 Å, 2.1 mm × 5 mm, 1.8 *µ*m, Waters). The column temperature was set to 30 °C. For the analysis of esculetin, fraxetin, scopoletin, and their glycosides, the gradient was linear from 0 min, 90% solvent A (0.1% (v/v) formic acid in 18 mΩ water [Milli-Q]) and 10% solvent B (0.1% (v/v) formic acid in acetonitrile), to 8 min, 20% solvent A and 80% solvent B. For the analysis of sideretin, the gradient was changed to linear within 7 min to 70% solvent A and 30% solvent B. In both methods, the solvent composition was then switched back to the initial conditions within 1 min, and then kept constant for another minute to equilibrate the column for the next run. In all cases, the flow rate was 0.4 mL min^−1^ and the injection volume was set to 1 *µ*L.

The eluted coumarins were sprayed into the MS using an electrospray ionization (ESI) source with Agilent Jet Stream technology (Agilent Technologies, United States). The sheath gas temperature was set to 300 °C. Mass spectra were acquired in positive and/or negative mode, with capillary voltages of 2 and 3 kV, respectively. For tandem mass spectrometry (MS/MS), a collision energy of 18 eV was found as optimal for all compounds. For data acquisition, a multiple reaction monitoring (MRM) MS experiment was set up. Supplemental Table S3 summarizes the MRM transitions and retention times for the different coumarins. The specific MRM transition for the compound 4-methyldaphnetin (193 > 147 *m*/*z* in pos. mode, 191 > 145 in neg. mode) was also measured. The UHPLC system was coupled to a Q Exactive Plus Mass Spectrometer (Thermo Fisher Scientific, Germany) equipped with a heated electrospray ionization source operating in both positive and negative ion modes. The source values were set in positive mode as follows: spray voltage 3.5 kV; capillary temperature 255 °C; S-lens RF level 40; aux gas heater temperature 400 °C; sheath gas flow rate 40; and aux gas flow rate 10. In negative mode, the spray voltage was set to 2.5 kV, the sheath gas flow rate to 47, and the aux gas flow rate to 11. A full MS ddMS2 experiment was performed for spectra acquisition. The resolution in the full scan was set to 70,000. For MS/MS experiments, a resolution of 17,500 and an NCE of 30 V was used. MS data were acquired and processed by the Trace Finder Software (v. 4.1., Thermo Fisher Scientific, Germany). During the study, the UPLC-MS method for coumarin analysis in root exudates was also transferred to an Orbitrap mass spectrometer. On this device, chromatographic separation was performed on a Vanquish UHPLC system (Thermo Fisher Scientific, Germany). Coumarin baseline separation was achieved using the same column and solvents as described above. The applied gradient was as follows: 0 to 1 min 10% solvent B; linear increase of solvent B to 20% at 5 min, to 30% at 8 min, to 65% at 9 min, and to 80% at 9.5 min; and 9.5 to 10 min 80% solvent B. Additional gradient steps to a total run time of 13 min were included to guarantee both column wash and equilibration. The column temperature was set to 30 °C and the flow rate to 0.4 mL min^−1^. The injection volume was 1 *µ*L.

A mixture of all coumarins with a final concentration of 0.4 *µ*g mL^−1^ was injected at least every 10 samples to ensure the stability of the measurement and validity of the calibration curve.

MS data were analyzed using MassHunter software (B.07.01, Agilent Technologies, United States). Data quantification was performed based on an external calibration curve. A stock solution with a final concentration of 0.8 *µ*g mL^−1^ of all coumarins analyzed in this study was prepared. The calibrationstandards were prepared by serial dilution of this stock solution (0.8 *µ*g mL^−1^, 0.7 *µ*g mL^−1^, 0.6 *µ*g mL^−1^, 0.5 *µ*g mL^−1^, 0.4 *µ*g mL^−1^, 0.3 *µ*g mL^−1^, 0.2 *µ*g mL^−1^, 0.1 *µ*g mL^−1^, 0.05 *µ*g mL^−1^, and 0.025 *µ*g mL^−1^). Each standard was measured 3 times and a linear regression curve was calculated.

### Cyclic voltammetry

An Metrohm-Autolab PGStat20 instrument with Metrohm VA stand 663 was used with a glassy carbon disc electrode (2 mm diameter) as working electrode, Ag/AgCl/KCl(3 M) as reference electrode, and Pt as counter electrode. Cyclic voltammograms were recorded at 100 mV per second in 20 mM ammonium acetate buffer pH 5.0 in the potential range of −500 mV up to +1,000 mV. Solutions were degassed with nitrogen gas for at least 300 s before measurement. Three potential scans were recorded for each coumarin and representative scans are shown.

### Gene expression analysis

Roots of 12 individual plants per biological replicate were pooled and directly frozen in liquid nitrogen. Total RNA was extracted from the homogenized samples using the NucleoSpin RNA Mini Kit (Machery-Nagel) according to the manufacturer's instruction. cDNA was synthesized from 0.5 to 1 *µ*g RNA by reverse transcription using the RevertAid First Strand cDNA synthesis Kit (Thermo Fisher Scientific) and oligo(dT) primer. A 10- or 20-times diluted cDNA sample was then used for reverse transcription quantitative PCR (RT-qPCR) analysis with the CFX384 Touch Real-Time PCR Detection System (Bio-Rad Laboratories) and the iQ SYBR Green Supermix (Bio-Rad Laboratories) using the primers listed in Supplemental Table S1. Recorded C_t_ values were exported from the Bio-Rad CFX Manager Software (Version 3.1, Bio-Rad Laboratories) and used for the calculation of PCR amplification efficiency and normalization factors using *ACTIN2* and *UBQ10* as reference genes. The PCR amplification efficiency was calculated according to previous instructions (Bustin et al. 2009). Normalization factors were calculated using geNORM (Vandesompele et al. 2002).

### Statistical analysis

Statistical analysis was performed using SigmaPlot 11.0 (Systat Software Inc.) and R (version 4.0.3). Pairwise comparisons were performed with 2-tailed Student's *t*-test and multiple comparisons with one-way ANOVA followed by a post hoc Tukey's test (*P* < 0.05). When any of the test assumptions could not be met, data were transformed. If data were still not normally distributed, the nonparametric Kruskal–Wallis (ANOVA on Ranks) test with post hoc Tukey's or Dunn's test (*P* < 0.05) or Student–Newman–Keuls (*P* < 0.05) was performed. When necessary, data were tested for any outliers using the two-sided Grubbs’ test with *P* < 0.05. Statistical data are provided in Supplemental Data Set S1.

### Accession numbers

Sequence data for the genes used in this study can be found in The Arabidopsis Information Resource (www.arabidopsis.org) under the following accession numbers: *FRO2*, AT1G01580; *IRT1*, AT4G19690; *F6′H1*, AT3G13610; *S8H*, AT3G12900; *CYP82C4*, AT4G31940; *PDR9*, AT3G53480; *MYB72*, AT1G56160; *FIT*, AT2G28160; *ACTIN2*, AT3G18780; and *UBQ10*, AT4G05320.

## Supplementary Material

koad279_Supplementary_Data

## Supplemental data

The following materials are available in the online version of this article.

**Supplemental Figure S1.** pH-dependent regulation of transcript levels of genes involved in coumarin biosynthesis and release.

**Supplemental Figure S2.** pH-dependent abundance and tissue-specific localization of F6′H1, S8H, and CYP82C4 in roots exposed to different external pH and buffer conditions in the presence of FeCl_3_.

**Supplemental Figure S3.** Coumarin concentration in roots of plants exposed to low Fe availability or Fe starvation at different external pH conditions.

**Supplemental Figure S4.** Coumarin concentration in roots of *s8h-1*, *cyp82C4-1*, and *pdr9-2* mutants.

**Supplemental Figure S5.** Coumarin exudation rates of *s8h-1*, *cyp82C4-1*, and *pdr9-2* mutants.

**Supplemental Figure S6.** Shoot biomass and chlorophyll concentration of wild-type and *myb72-1* plants grown under different pH conditions.

**Supplemental Figure S7.** Cyclic voltammograms of sideretin, fraxetin, esculetin, and scopoletin at acidic conditions.

**Supplemental Figure S8.** The effect of buffer strength on fraxetin-mediated Fe(III) mobilization and reduction.

**Supplemental Figure S9.** Effect of different pH conditions on the nutritional status of plants grown on agar plates with poorly available Fe.

**Supplemental Figure S10.** Effect of exogenous supply of coumarins on the maintenance of shoot growth and Fe concentrations of wild-type and *fro2* plants.

**Supplemental Figure S11.** Co-cultivation of *cyp82C4* alleviates leaf chlorosis and improves root growth of *fro2* plants at low pH.

**Supplemental Figure S12.** pH-dependent phenotype of the *fro2* mutant.

**Supplemental Figure S13.** Comparison of *fro2*, *irt1*, and *fro2 f6′h1-1* phenotypes and shoot Fe concentrations in slightly acidic soil substrate.

**Supplemental Figure S14.** The severe Fe-deficient phenotype of *fro2 f6′h1-1* double mutant plants is also observed under axenic conditions.

**Supplemental Figure S15.** Relative contribution of FRO2- and coumarin-mediated Fe mobilization depends on the pH of the soil substrate.

**Supplemental Figure S16.** Shoot growth and Fe concentration of *fro2 s8h-1* and *fro2 cyp82C4-1* double mutants on slightly acidic soil substrate.

**Supplemental Figure S17.** Coumarin exudation rate of *fro2 cyp82C4-1* plants.

**Supplemental Figure S18.** *fro2 cyp82C4-1* plants are more tolerant than *fro2 f6′h1-1* to high external pH.

**Supplemental Figure S19.** Molecular characterization of *fro2 f6′h1-1* double mutant.

**Supplemental Table S1.** Primers used in this study.

**Supplemental Table S2.** Settings used for the analysis of different mineral elements with sector-field ICP-MS in this study.

**Supplemental Table S3.** MRM transitions and retention times of coumarins analyzed in this study.

**Supplemental Data Set S1.** Statistical data.

## Dive Curated Terms

The following phenotypic, genotypic, and functional terms are of significance to the work described in this paper:

MYB72 Gramene: AT1G56160
MYB72 Araport: AT1G56160

AHA2 Gramene: AT4G30190
AHA2 Araport: AT4G30190

MLS Gramene: AT5G03860
MLS Araport: AT5G03860

FRO2 Gramene: AT1G01580
FRO2 Araport: AT1G01580

IRT1 Gramene: AT4G19690
IRT1 Araport: AT4G19690

PDR9 Gramene: AT3G53480
PDR9 Araport: AT3G53480

root AmiGo: PO:0009005

UBQ10 Gramene: At4g05320
UBQ10 Araport: At4g05320

fraxetin CHEBI: CHEBI:5169

NRAMP1 Gramene: AT1G80830
NRAMP1 Araport: AT1G80830

CYP82C4 Gramene: AT4G31940
CYP82C4 Araport: AT4G31940

ACT2 Gramene: AT3G18780
ACT2 Araport: AT3G18780

iron CHEBI: CHEBI:18248
