# Supplementary material for: A major role of coumarin-dependent ferric iron reduction in strategy I-type iron acquisition in Arabidopsis
Source: Plant Cell. 2023 Nov 28;36(3):642–64. doi: 10.1093/plcell/koad279 (PMC10896297; doi:10.1093/plcell/koad279)
Supplement: koad279_Supplementary_Data [file koad279_supplementary_data.zip › tpc.23.00242Supplemental Figures and Tables.pdf]

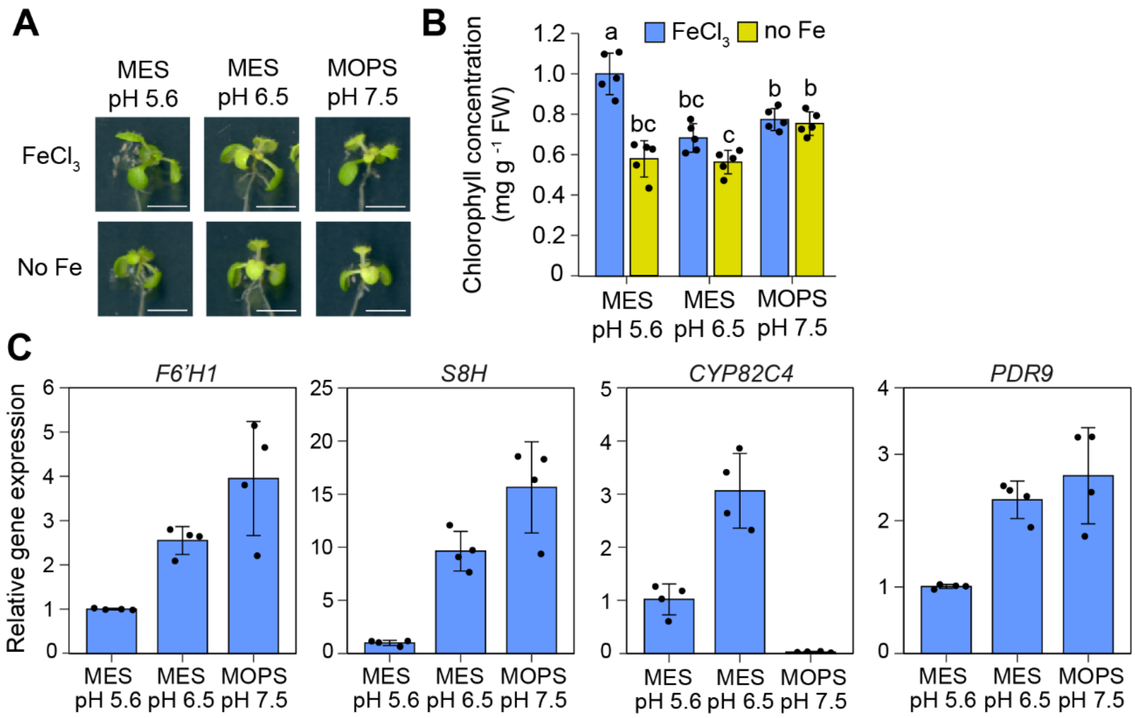

Supplemental Figure S1. pH-dependent regulation of transcript levels of genes involved in coumarin biosynthesis and release. Supports Figure 1.

(A-B) Appearance (A), leaf chlorophyll concentration (B) of wild-type (Col-0) plants grown for 4 d under different Fe-limiting conditions and external pHs. Plants were pre-cultured for 10 d on half-strength MS medium with 40 µM FeEDTA at pH 5.6 and then transferred to half-strength MS medium with 20 µM $FeCl_3$ ($FeCl_3$) or without added Fe and 15 µM ferrozine (no Fe) buffered with MES to pH 5.6 or pH 6.5, or MOPS to pH 7.5. Bars indicate means ± s.d. (n = 5 biological replicates composed of 4 shoots each) and different letters indicate significant differences ($P < 0.05$) according to one-way ANOVA with post-hoc Tukey's test at $p<0.05$. (C) Relative transcript levels of *F6'H1*, *S8H*, *CYP82C4*, and *PDR9* in roots of wild-type (Col-0) plants grown for 4 d without added Fe. After 10 d of pre-culture on half-strength MS medium with 40 µM FeEDTA at pH 5.6, plants were transferred to half-strength MS medium without added Fe plus 15 µM ferrozine at the indicated pH conditions. Relative transcript levels were normalized to *UBQ10* and *ACT2*. Bars indicate means ± s.d. (n = 4 biological replicates composed of pooled roots of 12 plants each). Scale bars, 0.5 cm.

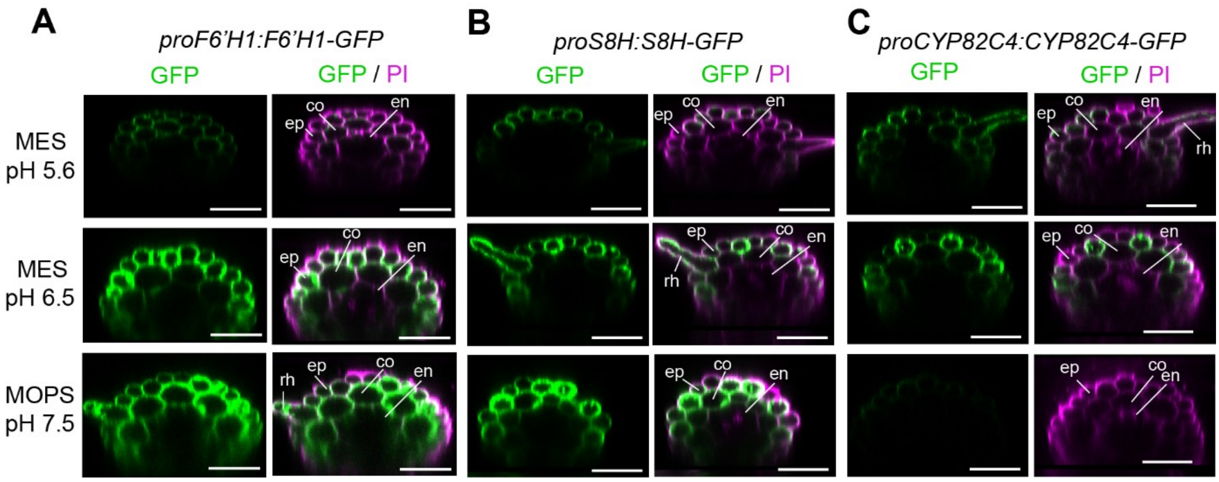

Supplemental Figure S2. pH-dependent abundance and tissue-specific localization of F6'H1, S8H and CYP82C4 in roots exposed to different external pH and buffer conditions in the presence of $FeCl_3$. Supports Figure 1.

(A-C) Green fluorescent protein (GFP, green) and merged GFP and propidium iodide (PI, magenta) signals detected in the root hair zone of *proF6'H1:F6'H1-GFP* (A), *proS8H:S8H-GFP* (B) and *proCYP82C4:CYP82C4-GFP* (C) translation fusion lines. Plants were pre-cultured for 10 d on half-strength MS medium with 40 µM FeEDTA at pH 5.6 and then transferred to half-strength MS medium with 20 µM $FeCl_3$ and buffered with either MES to pH 5.6 or pH 6.5, or MOPS to pH 7.5. Images were taken 4 days after transfer to the indicated conditions. Shown are transverse sections reconstituted from Z-stacks. Root tissue layers are labeled as ep: epidermis, co: cortex, en: endodermis and rh, root hair. Scale bars = 50 µm.

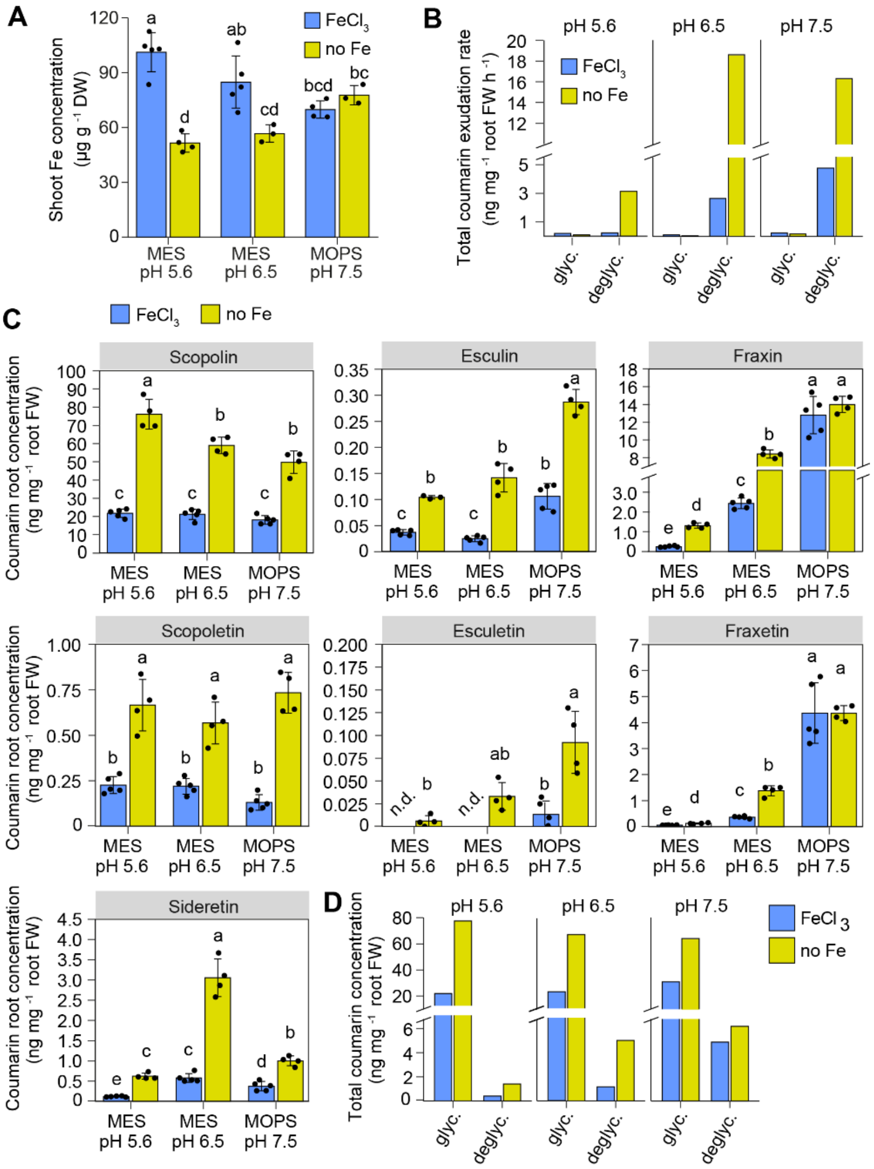

Supplemental Figure S3. Coumarin concentration in root extracts of plants exposed to low Fe availability or Fe starvation at different external pH conditions. Supports Figure 2.
(A-B) Shoot Fe concentration (A) and total exudation rates of glycosylated (glyc. = scopolin + esculin + fraxin) and deglycosylated (deglyc. = scopoletin + esculetin + fraxetin + sideretin) coumarins (B) in *A. thaliana* (Col-0) plants grown for 4 d under different Fe-limiting conditions and external pHs. Bars represent means ± s.d. (n = 3-5 biological replicates composed of 4 shoots each in A, and 4-5 biological replicates composed of pooled exudates from 140 plants each in B). (C) Concentration of the indicated coumarins in root extracts of wild-type (Col-0) plants grown for 4 d under different Fe-limiting conditions and external pHs. Sideretin represents the oxidized and reduced forms combined. Bars represent means ± s.d. (n = 4-5 biological replicates composed of pooled exudates from 140 plants each). (D) Total root coumarin concentration as sum of glycosylated (glyc. = scopolin + esculin + fraxin) and deglycosylated (deglyc. = scopoletin + esculetin + fraxetin + sideretin) coumarins. In A-D, plants were pre-cultured for 10 d on half-strength MS medium with 40 µM FeEDTA at pH 5.6 and then transferred to half-strength MS medium with 20 µM $FeCl_3$ ($FeCl_3$) or without added Fe and 15 µM ferrozine (no Fe) buffered with MES to pH 5.6 or pH 6.5, or MOPS to pH 7.5. After 4 d, root exudates were collected for 6 h on water adjusted to the respective pH and buffered with either MES or MOPS. Different letters indicate significant differences ($P < 0.05$) according to one-way ANOVA with post-hoc Tukey's test or ANOVA on ranks with post-hoc Dunn's test. FW, fresh weight; DW, dry weight.

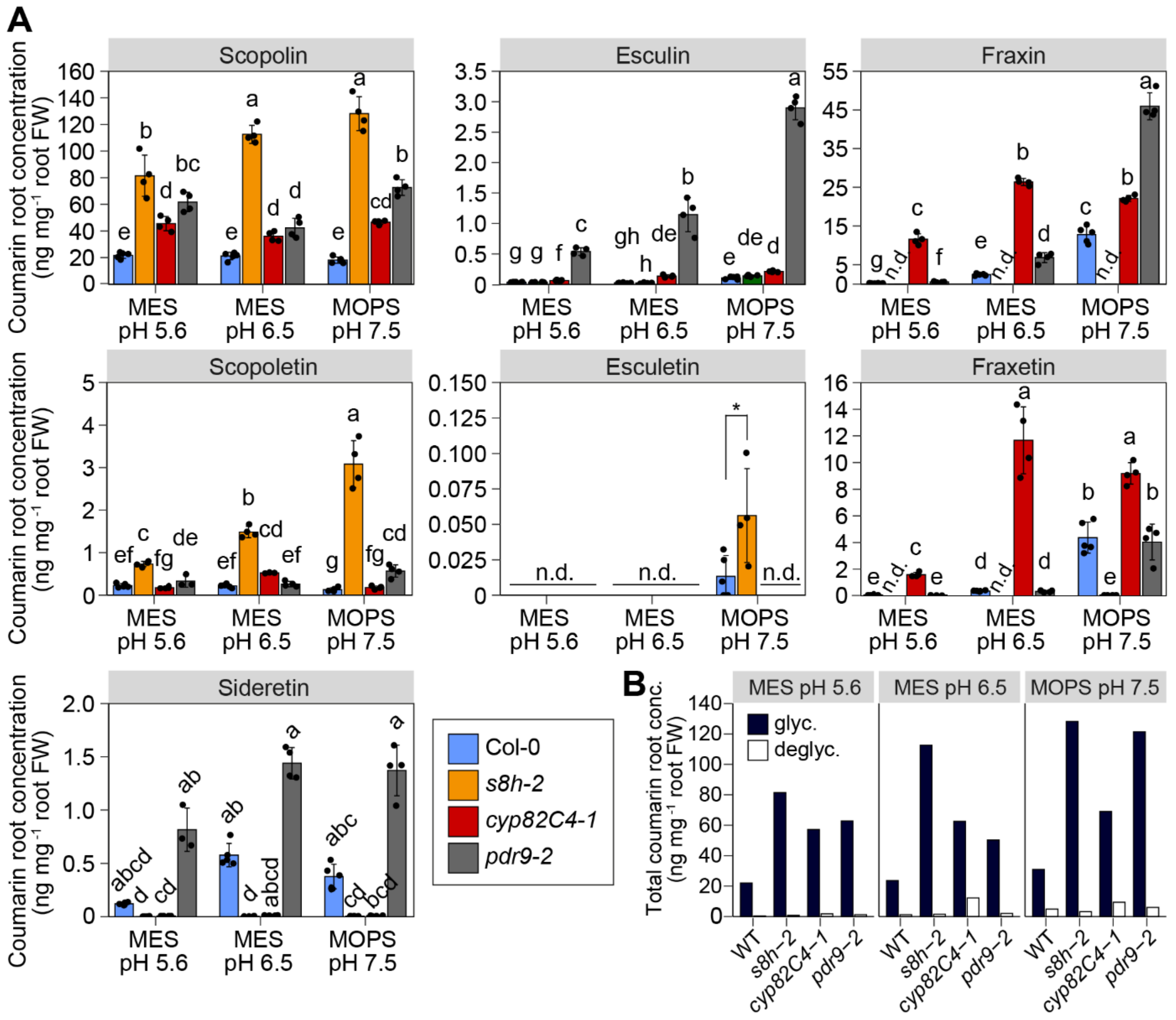

Supplemental Figure S4. Coumarin concentration in roots of *s8h-1*, *cyp82C4-1* and *pdr9-2* mutants. Supports Figure 2.

(A) Concentration of indicated coumarin in roots of wild-type (Col-0), *s8h-2*, *cyp82C4-1* and *pdr9-2* plants grown for 4 d under different conditions of low Fe-availability. After 10 d pre-culture on half-strength MS medium with 40 µM FeEDTA at pH 5.6, plants were transferred to half-strength MS medium with 20 µM $FeCl_3$ buffered with either MES to pH 5.6 or 6.5, or MOPS to pH 7.5. Sideretin represents the oxidized and reduced forms combined. Root coumarin concentrations for wild-type plants are also shown in Suppl. Fig. 3 (experiment performed at the same time) and root fraxin concentration is also presented in Fig. 2B. Bars represent means ± s.d (n = 3-5 biological replicates composed of roots of 140 plants each). Different letters indicate significant differences (P < 0.05) according to one-way ANOVA with post-hoc Tukey's test (scopoletin, esculin, fraxin, scopoletin, esculetin and fraxetin) or ANOVA on ranks with post-hoc Dunn's test (sideretin). (B) Total root coumarin concentration as sum of glycosylated (glyc. = scopolin + esculin + fraxin) and deglycosylated (deglyc. = scopoletin + esculetin + fraxetin + sideretin) coumarins. FW, fresh weight.

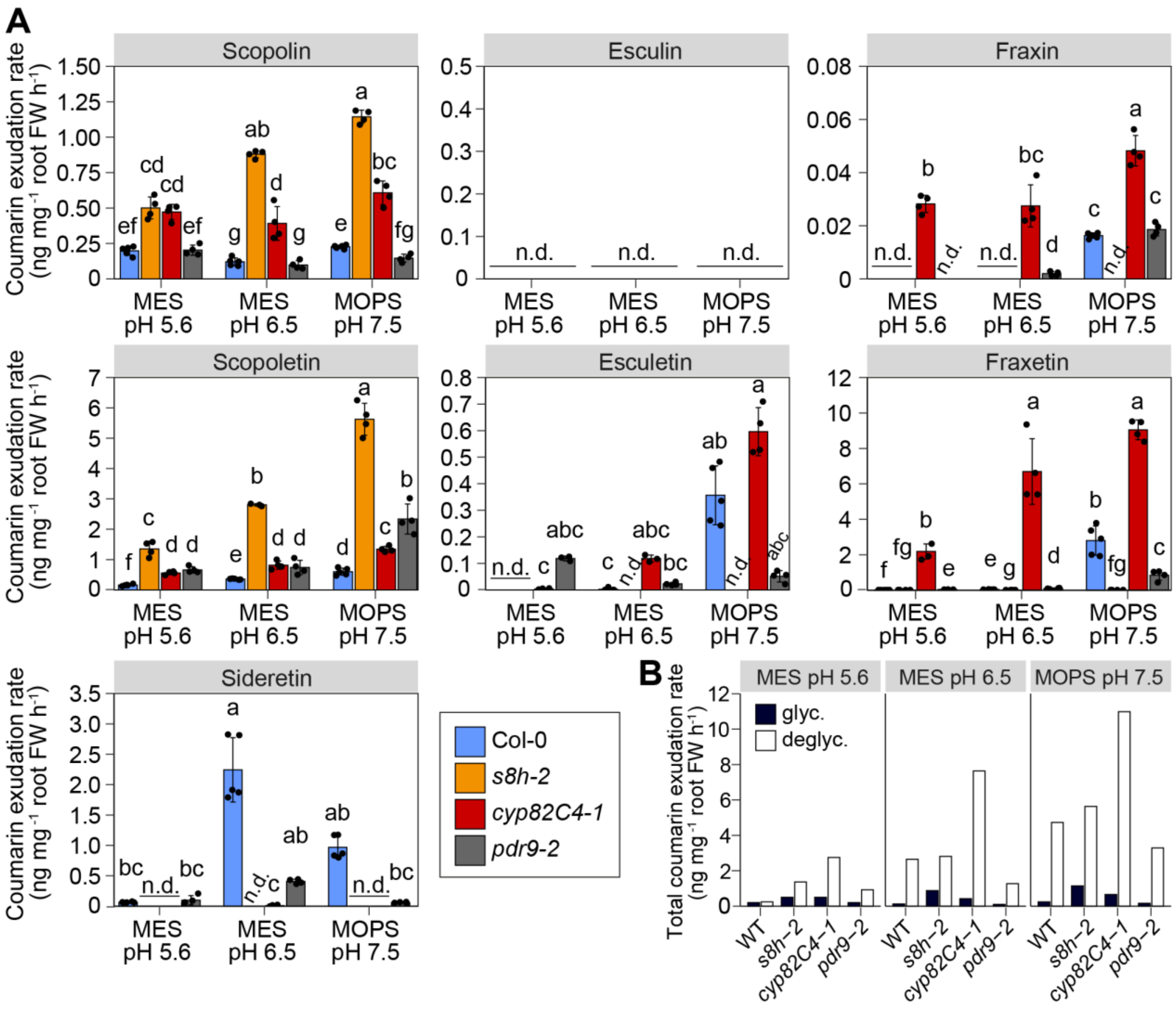

Supplemental Figure S5. Coumarin exudation rates of *s8h-1*, *cyp82C4-1* and *pdr9-2* mutants. Supports Figure 2.

(A) Exudation rates of indicated coumarins from roots of wild-type (Col-0), *s8h-2*, *cyp82C4-1*, and *pdr9-2* plants grown for 4 d under different conditions of low Fe-availability. After 10 d pre-culture on half-strength MS medium with 40 µM FeEDTA at pH 5.6, plants were transferred to half-strength MS medium with 20 µM $FeCl_3$ buffered with MES to pH 5.6 or 6.5, or MOPS to pH 7.5. Root exudates were collected for 6 h on water adjusted to the respective pH and buffered with either MES or MOPS. Sideretin represents the oxidized and reduced forms combined. Coumarin exudation rates for wild-type plants are also shown in Fig. 2A (experiment performed at the same time) and for the indicated mutants in Fig. 2B. Bars represent means ± s.d (n = 3-5 biological replicates composed of pooled exudates from 140 plants each). Different letters indicate significant differences (*P* < 0.05) according to one-way ANOVA with post-hoc Tukey's test (scopolin, scopoletin, fraxetin) or ANOVA on ranks with post-hoc Dunn's test (fraxin, esculetin, sideretin). (B) Total exudation rates of glycosylated (glyc. = scopolin + esculin + fraxin) and deglycosylated (deglyc. = scopoletin + esculetin + fraxetin + sideretin) coumarins. FW, fresh weight.

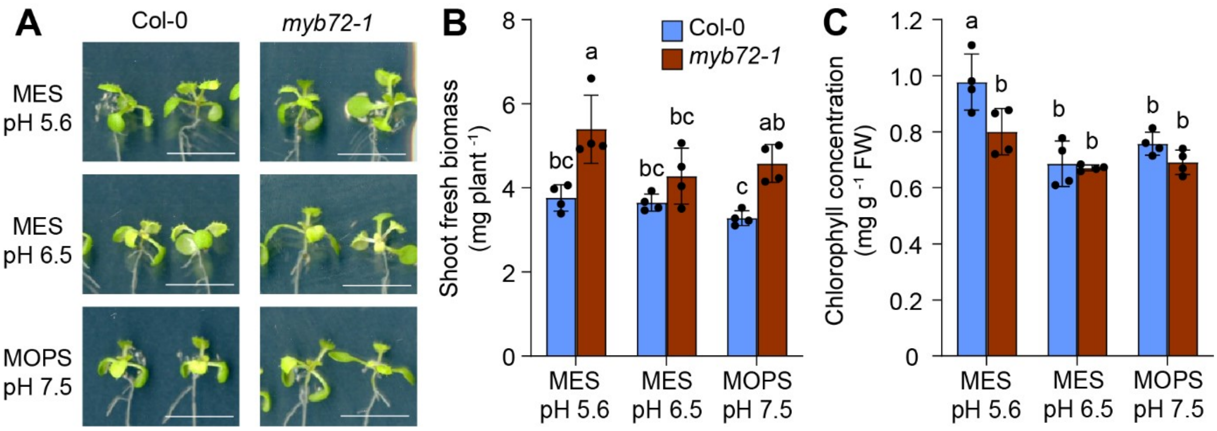

Supplemental Figure S6. Shoot biomass and chlorophyll concentration of wild-type and *myb72-1* plants grown under different pH conditions. Supports Figure 3.

(A-D) Appearance (A), fresh biomass (B) and chlorophyll concentration (C) of shoots of Col-0 and *myb72-1* plants assessed 4 days after transfer to the indicated conditions. Plants were pre-cultured for 10 d on half-strength MS medium with 40 µM FeEDTA at pH 5.6 and then transferred to half-strength MS medium with 20 µM $FeCl_3$ ($FeCl_3$) buffered with MES to pH 5.6 or pH 6.5, or with MOPS to pH 7.5. Bars represent means ± s.d (n = 4 biological replicates composed of 5 shoots each). Different letters indicate significant differences ($P < 0.05$) according to one-way ANOVA with post-hoc Tukey's test. FW, fresh weight. Scale bars, 1 cm.

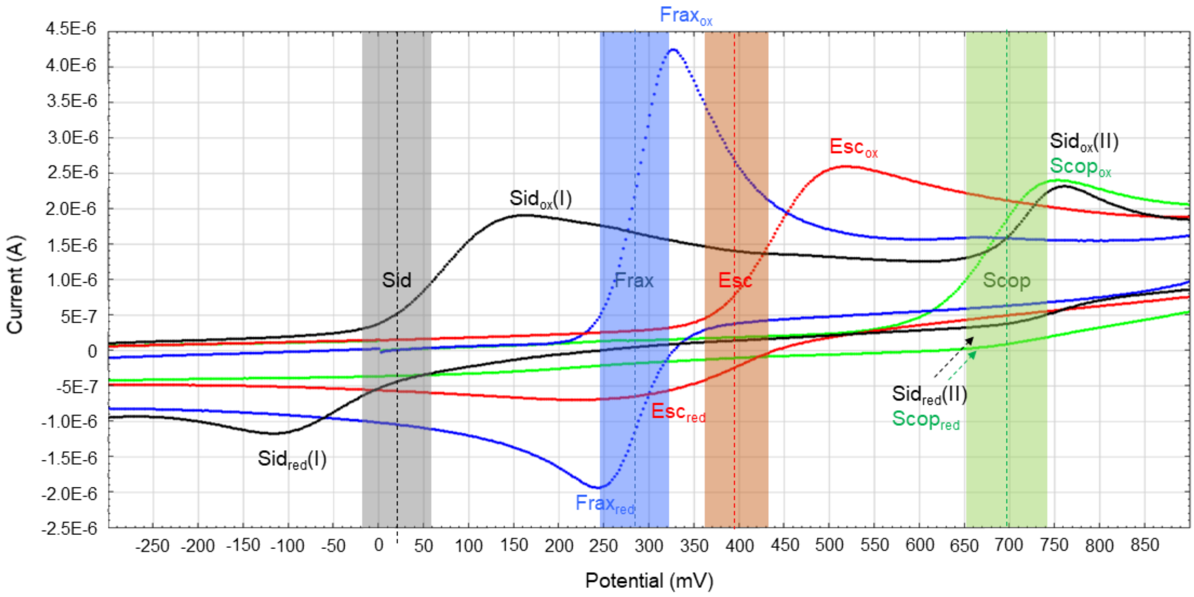

Supplemental Figure S7. Cyclic voltammograms of sideretin, fraxetin, esculetin, and scopoletin at acidic conditions. Supports Figure 4.

Cyclic voltammogram recordings were made at pH 5.0 in 20 mM ammonium acetate with GC electrode (with 3 mol L^{-1} Ag/AgCl/KCl as reference) and 100 mV/s scan rate. The colored areas highlight the mid-point potentials of the different coumarins. Scop, scopoletin; Esc, esculetin; Frax, fraxetin; Sid, sideretin; red, reduced; ox, oxidized.

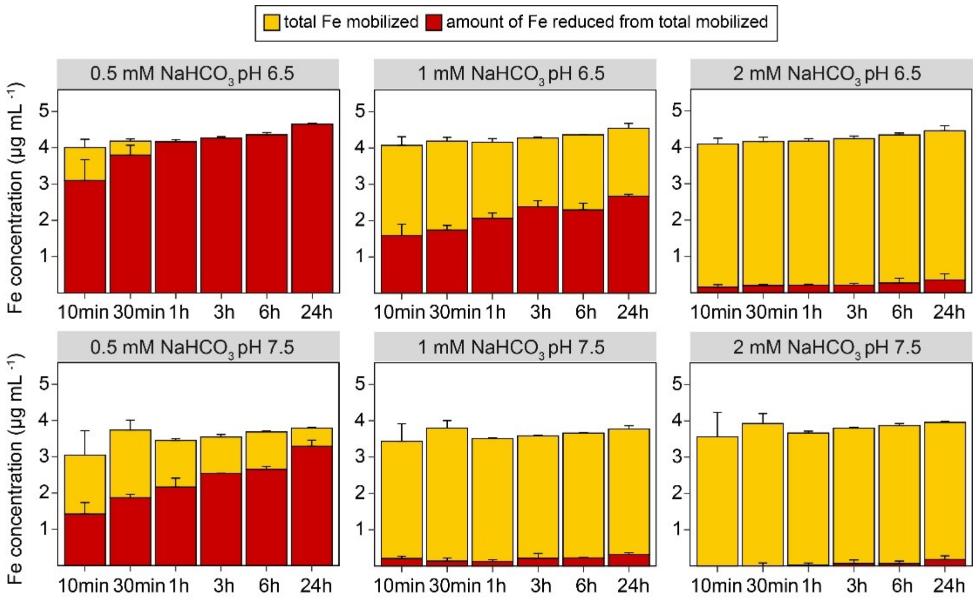

Supplemental Figure S8. The effect of buffer strength on fraxetin-mediated Fe(III) mobilization and reduction. Supports Figure 4.

Time-dependent *in vitro* Fe(III) mobilization (yellow) and reduction (red) by fraxetin at different pH and $NaHCO_3$ concentrations. Fraxetin was incubated in darkness with freshly precipitated Fe (supplied as $FeCl_3$) in 0.5 mM, 1 mM, or 2 mM sodium bicarbonate ($NaHCO_3$) buffered to pH 6.5 or pH 7.5. Aliquots were taken 10 min, 30 min, 1 h, 3 h, 6 h, and 24 h after starting the reaction. The amount of total mobilized Fe was determined by ICP-MS and the amount of reduced Fe was assessed spectrophotometrically on the basis of the formation of Fe(II)-ferrozine complexes. The upper end of the y-axis represents the total amount of Fe used in the experiments. Bars represent means ± s.d. (n = 3 independent reactions).

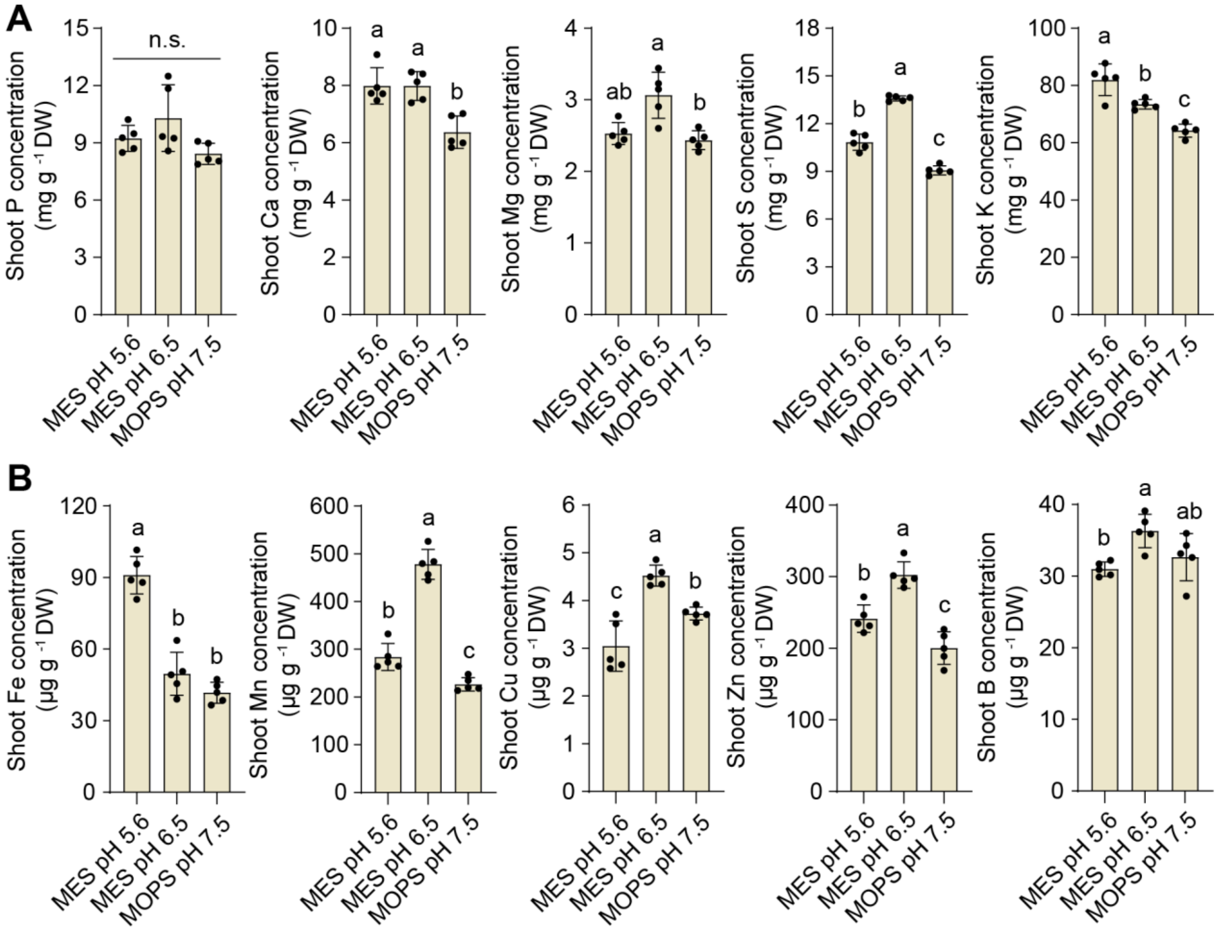

Supplemental Figure S9. Effect of different pH conditions on the nutritional status of plants grown on agar plates with poorly available Fe. Supports Figure 5.

(A-B) Concentration of macronutrients (A) and micronutrients (B) in shoots of *A. thaliana* (Col-*gl1*) plants grown for 6 d in solid agar medium under the indicated pH conditions. After 10 d pre-culture on solid half-strength MS medium with 40 µM FeEDTA at pH 5.6, seedlings were transferred to half-strength MS medium with 20 µM $FeCl_3$ buffered with MES to pH 5.6 or 6.5, or MOPS to pH 7.5. Bars represent means ± s.d (n = 5 biological replicates containing 4 pooled shoots each). Shoot Cu concentration were below critical levels, as it is common when seedlings are grown on half-strength MS medium. Different letters indicate significant differences ($P < 0.05$) according to one-way ANOVA with post-hoc Tukey's test. DW, dry weight.

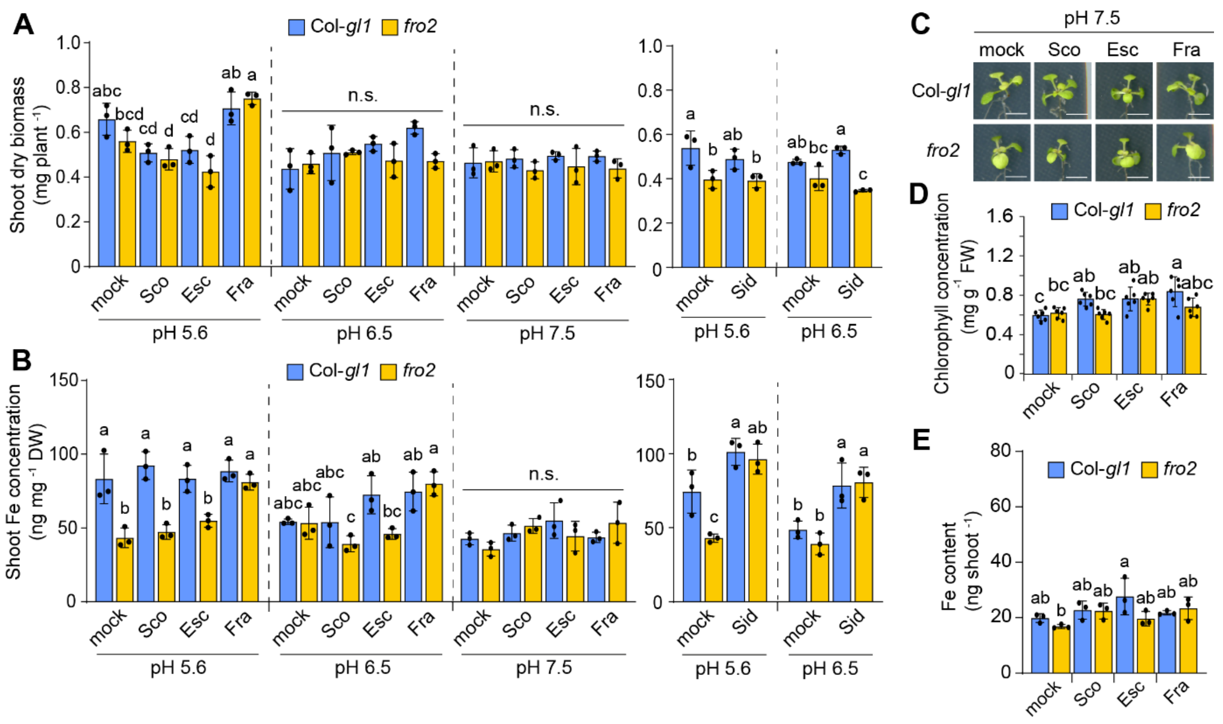

Supplemental Figure S10. Effect of exogenous supply of coumarins on the maintenance of shoot growth and Fe concentrations of wild-type and *fro2* plants. Supports Figure 5.

(A-B), Dry biomass (A) and Fe concentration of shoots (B) of wild-type (Col-*gl1*) and *fro2* plants after 6 d of cultivation in solid agar medium supplied with different coumarins. Ten-day-old seedlings pre-cultured on half-strength MS medium with 40 µM Fe-EDTA at pH 5.6 were transferred to half-strength MS medium with 20 µM $FeCl_3$ buffered with MES to pH 5.6 or pH 6.5, or MOPS to pH 7.5. The medium was supplied with scopoletin (Sco), esculetin (Esc), fraxetin (Fra), sideretin (Sid) or only the solvent (mock). Bars represent means ± s.d. (n = 3 biological replicates composed of 4 pooled shoots each). Different letters within each pH condition indicate significant differences ($P < 0.05$) according to one-way ANOVA with post-hoc Tukey's test. (C-E), Plant appearance (C), shoot chlorophyll concentration (D) and shoot Fe content (E) of wild-type (Col-*gl1*) and *fro2* plants after 6 d of cultivation in solid agar medium supplied with different coumarins. Ten-day-old seedlings pre-cultured on half-strength MS medium with 40 µM Fe-EDTA at pH 5.6 were transferred to half-strength MS medium with 20 µM $FeCl_3$ buffered with MOPS to pH 7.5. The medium was supplied with scopoletin (Sco), esculetin (Esc), fraxetin (Fra), or only the solvent (mock). Bars represent means ± s.d. (n = 6 biological replicates in D or n = 3 biological replicates composed of 4 pooled shoots each). Different letters within each pH condition indicate significant differences ($P < 0.05$) according to one-way ANOVA with post-hoc Tukey's test. FW, fresh weight; DW, dry weight. Scale bars, 0.5 cm.

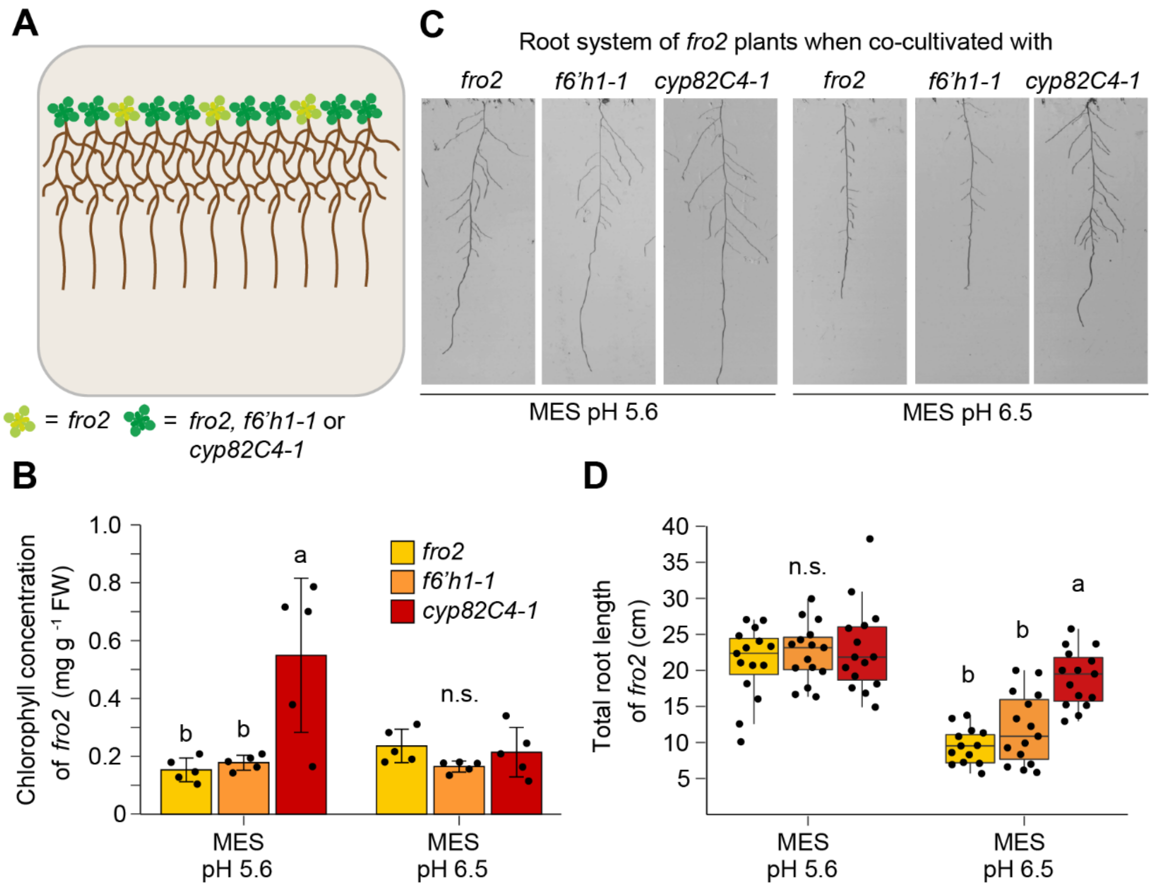

Supplemental Figure S11. Co-cultivation of *cyp82C4* alleviates leaf chlorosis and improves root growth of *fro2* plants at low pH. Supports Figure 5.

(A-D) Schematic representation of the experiment (A), leaf chlorophyll concentration (B), root system appearance (C), and total root length (D) of *fro2* plants co-cultivated either with itself or with *f6'h1-1* or *cyp82C4-1*. Plant were pre-cultured on half-strength MS medium with 40 µM Fe-EDTA at pH 5.6 for 10 d and then transferred to half-strength MS with 20 µM $FeCl_3$ buffered with MES to pH 5.6 or pH 6.5 for another 11 d. Bars represent means ± s.d. (n = 5 biological replicates composed of 3 pooled shoots). Box plots horizontal lines show medians (n = 15 independent roots), box limits indicate the 25th and 75th percentiles and whiskers extend to 5th and 95th percentiles. Different letters indicate significant differences ($P < 0.05$) according to one-way ANOVA with post-hoc Tukey's test. FW, fresh weight.

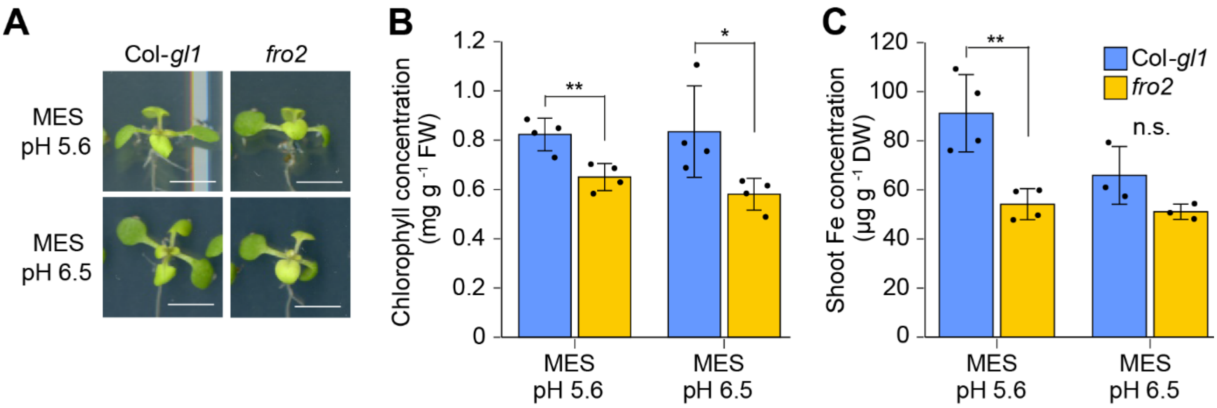

Supplemental Figure S12. pH-dependent phenotype of the *fro2* mutant. Supports Figure 6.
(A-C) Appearance (A), leaf chlorophyll concentration (B), and shoot Fe concentration (C) of wild-type (Col-*gl1*) and *fro2* plants pre-cultured on half-strength MS medium with 40 µM Fe-EDTA at pH 5.6 for 10 d and then transferred to half-strength MS medium without added Fe plus 15 µM ferrozine, and buffered with MES to pH 5.6 or pH 6.5 for 4 d. Bars represent means ± s.d. (n = 3-4 biological replicates composed of 4 pooled shoots each). Significant differences according to Student's *t*-test between the indicated pairwise comparisons are indicated with asterisks (* $P < 0.05$ and ** $P < 0.01$). n.s., not significant ($P > 0.05$). FW, fresh weight; DW, dry weight. Scale bars, 0.5 cm.

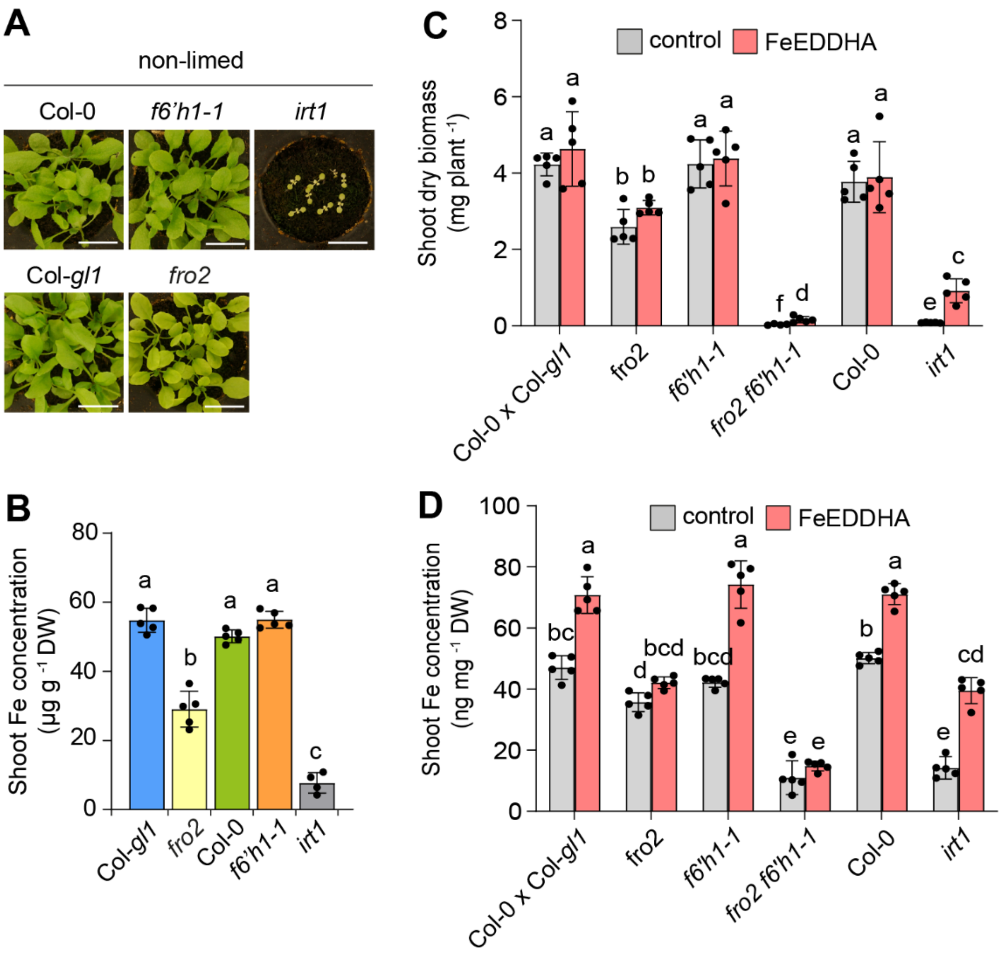

Supplemental Figure S13. Comparison of *fro2, irt1* and *fro2 f6'h1-1* phenotypes and shoot Fe concentrations in slightly acidic soil substrate. Supports Figure 7.

(A-B) Appearance (A) and shoot Fe concentration (B) of *f6'h1-1*, *irt1-1*, *fro2* and respective wild-type (Col-0 and Col-*gl1*) plants grown for 19 d on non-limed substrate. The pH measured at the beginning of the experiment was 5.6. Bars represent means ± s.d. (n = 4-5 biological replicates composed of 4-8 pooled shoots each). Different letters indicate significant differences ($P < 0.05$) according to one-way ANOVA with post-hoc Tukey's test. Scale bars, 2 cm. (C-D) Shoot biomass (C) and Fe concentration of the indicated genotypes after 19 d of growth on non-limed substrate supplemented (FeEDDHA) or not (control) with Fe(III)-EDDHA. Bars represent means ± s.d. (n = 5 biological replicates composed of 4-8 pooled shoots each) and different letters represent significant differences ($P < 0.05$) according to one-way ANOVA with post-hoc Tukey's test (B, D) or one-way ANOVA on ranks with post-hoc Student-Newman-Keuls test (C). DW, dry weight.

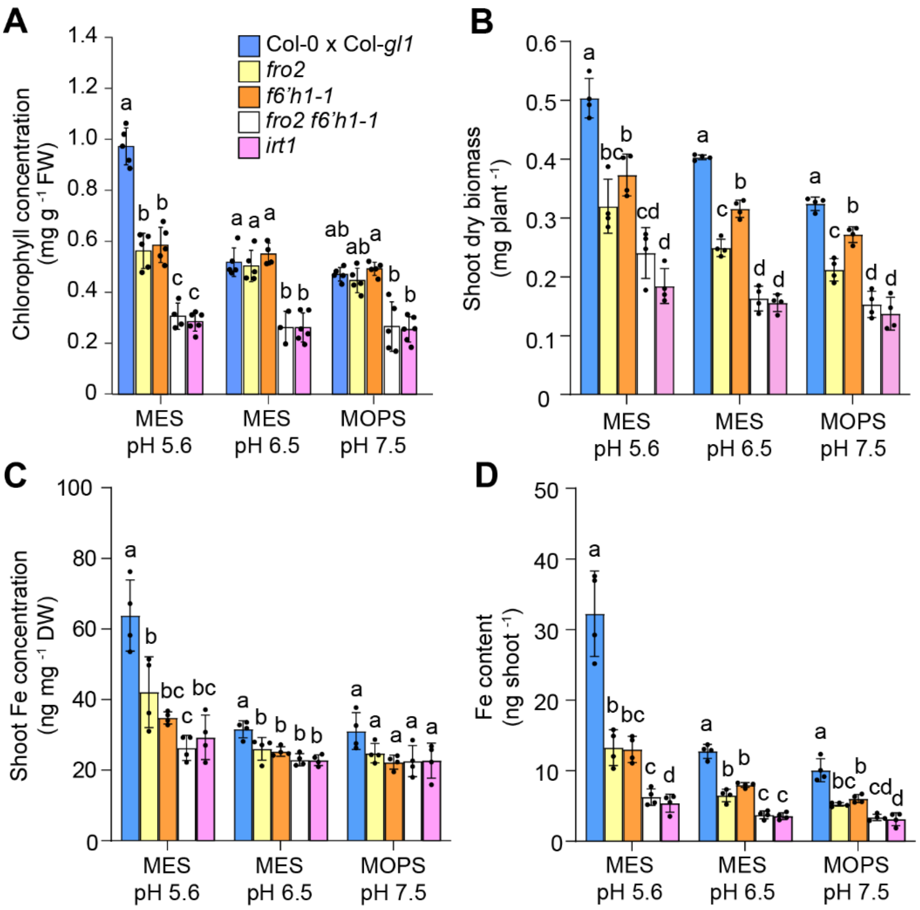

Supplemental Figure S14. The severe Fe-deficient phenotype of *fro2 f6'h1-1* double mutant plants is also observed under axenic conditions. Supports Figure 7.

(A-D) Shoot chlorophyll concentration (A), shoot dry biomass (B), shoot Fe concentration (C) and shoot Fe content (D) of wild-type (Col-0 x Col-*gl1*), *fro2*, *f6'h1-1*, *fro2 f6'h1-1* double mutant, and *irt1-1* plants after 6 d of cultivation under different low Fe availability conditions. Ten-day-old seedlings pre-cultured on half-strength MS medium with 40 µM Fe-EDTA at pH 5.6 were transferred to half-strength MS with 20 µM $FeCl_3$ buffered with either MES to pH 5.6 or pH 6.5, or MOPS to pH 7.5. Bars represent means ± s.d. (n = 3-6 biological replicates in A, and n = 4 biological replicates in B-D consisting of 4 pooled shoots each). Different letters within each pH condition indicate significant differences ($P < 0.05$) according to one-way ANOVA with post-hoc Tukey's test. FW, fresh weight; DW, dry weight.

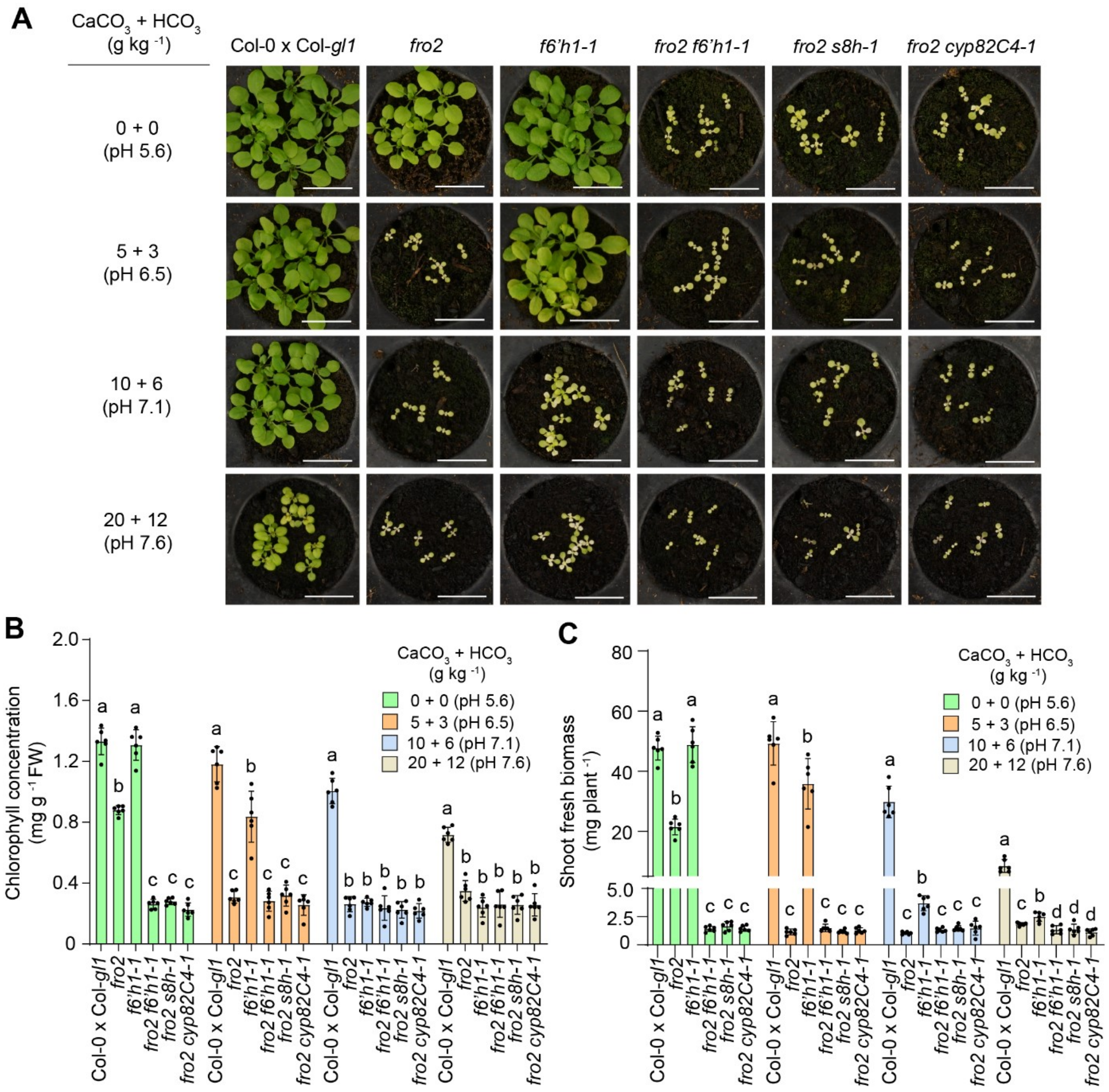

Supplemental Figure S15. The relative contribution of FRO2- and coumarin-mediated Fe mobilization depends on the pH of the soil substrate. Supports Figure 7.

(A-C) Plant appearance (A), leaf chlorophyll concentration (B) and shoot fresh biomass (C) of wild-type (Col-0 x Col-*gl1*), *fro2*, *f6'h1-1* and the indicated double mutants after 19 days of cultivation on non-limed substrate (pH 5.6) or on substrates limed with different concentrations of calcium carbonate ($CaCO_3$) and bicarbonate (HCO_3). The pH at the beginning of the experiment for the different substrate batches is indicated. Bars represent means ± s.d. (n = 6 biological replicates consisting of 3 pooled shoots each). Different letters represent significant differences ($P < 0.05$) according to one-way ANOVA with post-hoc Tukey's test (B) or one-way ANOVA on ranks with post-hoc Student-Newman-Keuls test (C). FW, fresh weight. Scale bars, 2 cm.

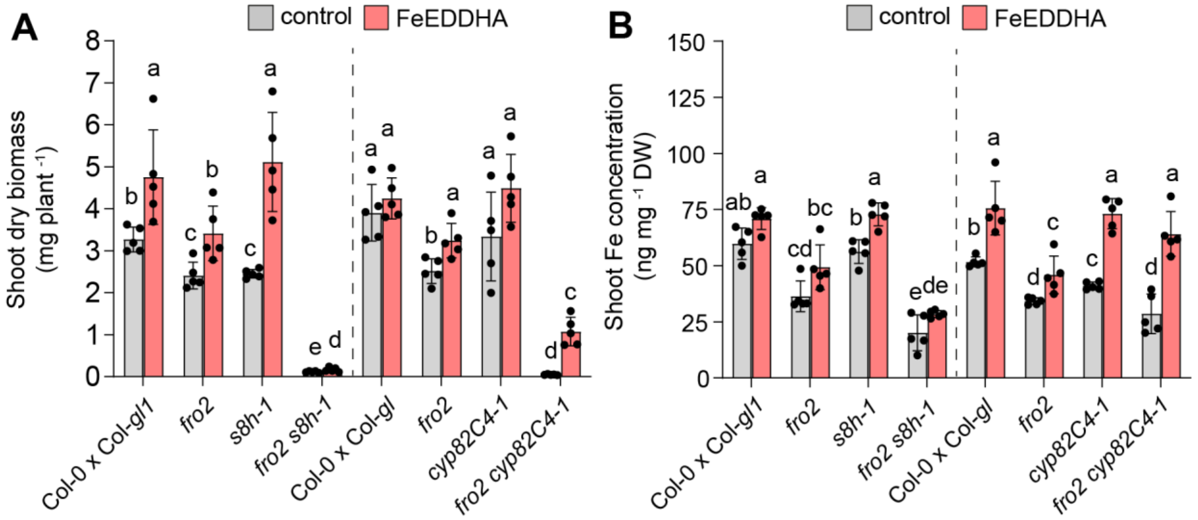

Supplemental Figure S16. Shoot growth and Fe concentration of *fro2 s8h-1* and *fro2 cyp82C4-1* double mutants on slightly acidic soil substrate. Supports Figure 8.

Dry biomass (A) and Fe concentration (B) of indicated genotypes after 19 days of growth on non-limed substrate (pH ~5.6) supplemented or not three times per week with Fe(III)-EDDHA. Bars represent means ± s.d. (n = 5 biological replicates consisting of 5 pooled shoots each). Different letters within each genotype group separated by dashed lines represent significant differences ($P < 0.05$) according to one-way ANOVA with post-hoc Tukey's test (B left) or one-way ANOVA on ranks with post-hoc Student-Newman-Keuls test (A left and right, and B right). DW, dry weight.

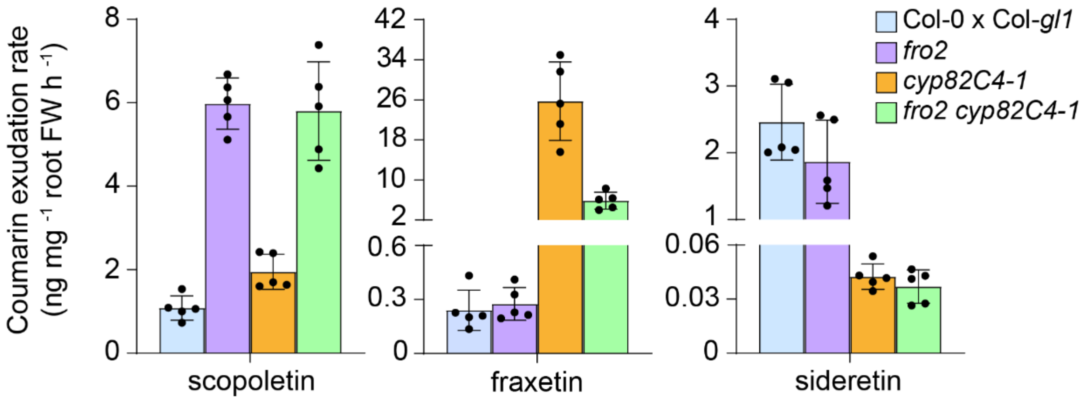

Supplemental Figure S17. Coumarin exudation rate of *fro2 cyp82C4-1* plants. Supports Figure 8.

Total exudation rates of scopoletin, fraxetin and sideretin of wild-type (Col-0 x Col-*gl1*), *fro2*, *cyp82C4-1* and *fro2 cyp82C4-1* double mutant. Plants were pre-cultured for 10 d on half-strength MS medium with 40 µM FeEDTA at pH 5.6 and then transferred to half-strength MS medium without added Fe (+15 µM ferrozine) buffered with MES to pH 5.6. After 4 d, root exudates were collected for 6 h on water adjusted to the respective pH and buffered with MES. Sideretin represents the oxidized and reduced forms combined. Bars represent means ± s.d. (n = 5 biological replicates composed of pooled exudates collected from 140 plants each). FW, fresh weight.

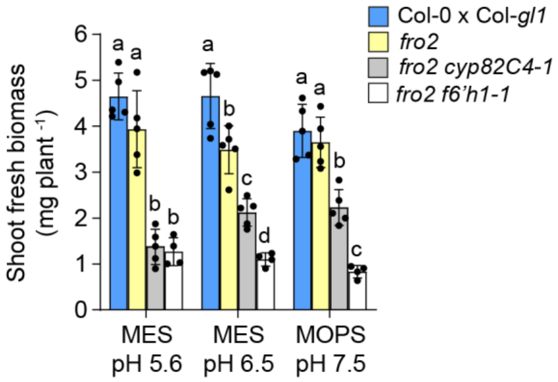

Supplemental Figure S18. *fro2 cyp82C4-1* plants are more tolerant than *fro2 f6'h1-1* to high external pH. Supports Figure 8.

Shoot fresh biomass of the indicated genotypes after 6 d of cultivation under different external pH conditions in solid agar medium. Ten-d-old seedlings pre-cultured on half-strength MS medium with 40 µM Fe-EDTA at pH 5.6 were transferred to half-strength MS with 20 µM $FeCl_3$ buffered with either MES to pH 5.6 or pH 6.5, or MOPS to pH 7.5. Bars represent means ± s.d. (n = 4-5 biological replicates composed of 4 pooled shoots each). Different letters within each pH condition indicate significant differences ($P < 0.05$) according to one-way ANOVA with post-hoc Tukey's test.

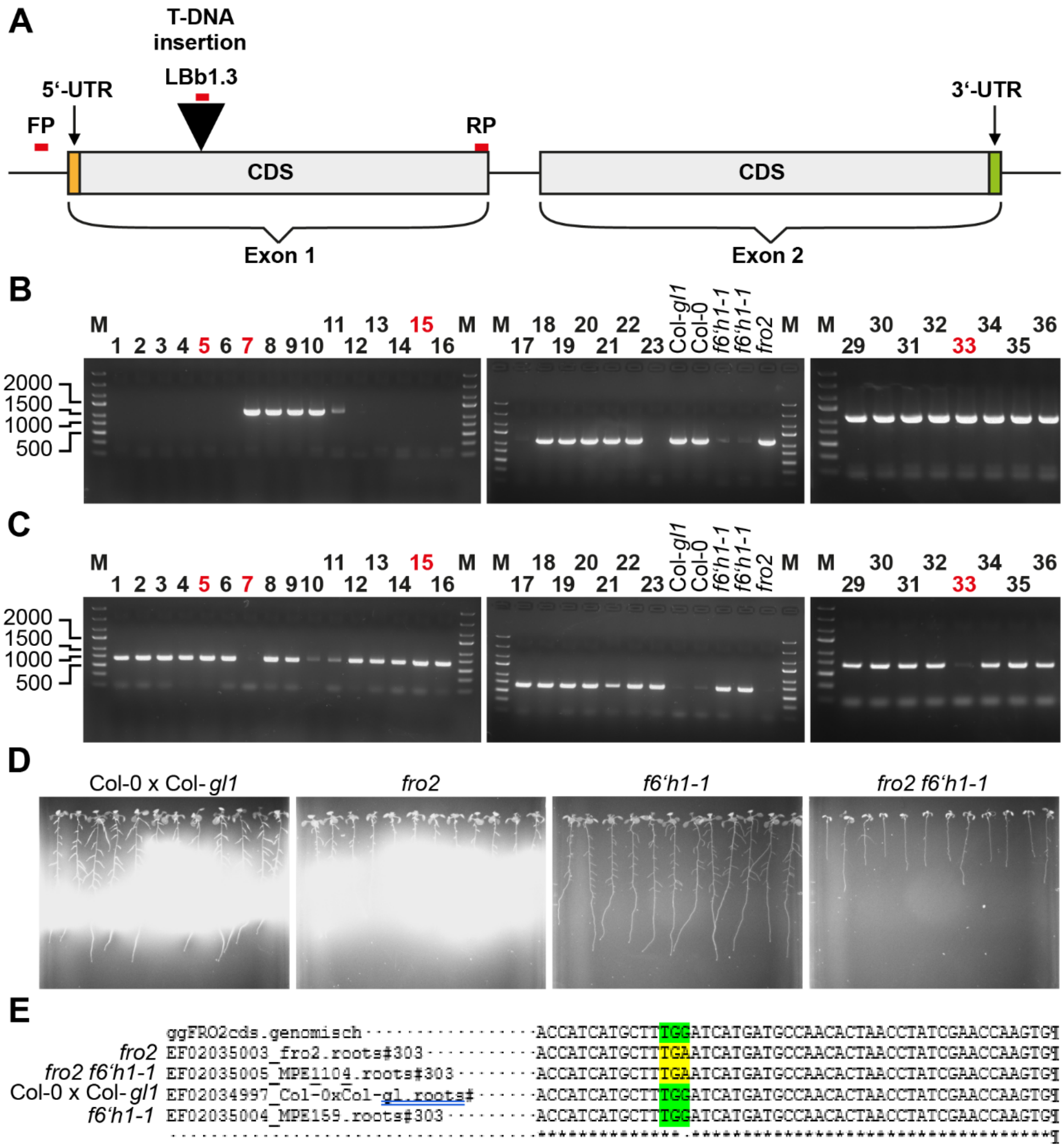

Supplemental Figure S19. Molecular characterization of *fro2 f6'h1-1* double mutant. Supports Material and Methods.

(A) Schematic representation of the *F6'H1* gene structure comprising two exons (exon 1: 701 bp; exon 2: 769 bp) separated by an 83-bp-long intron. In *f6'h1-1*, the T-DNA is located in the first exon. Primers used for PCR are indicated as red lines. UTR: untranslated region; FP: forward primer; LBb1.3: T-DNA specific primer for SALK collection, RP: reverse primer. (B-C) Isolation of wild-type (Col-0 x Col-*gl1*), single mutant *fro2* and *f6'h1-1* mutants, and double mutant plants (*fro2 f6'h1-1*) from a F2 population obtained after crossing *fro2* and *f6'h1-1* single mutants. PCR results for wild-type *F6'H1* locus (B) and for the presence of the expected T-DNA insertion (C). Red numbers indicate homozygous plants that were selected for further experiments (33, wild-type; 7, *fro2*; 15, *f6'h1-1*; 5, *fro2 f6'h1-1*). (D) Coumarin-dependent UV fluorescence (365 nm) in roots of wild-type, single mutants, and double mutant plants grown under Fe-limiting conditions as evidence for the absence of coumarins in *f6'h1-1* and *fro2 f6'h1-1* plants. (E) The G→A mutation at position 126 in the first exon of *FRO2* was verified by sequencing.

Supplemental Table S1. Primers used in this study. Supports Material and Methods.

**Primers used for genotyping**		
AGI ID	Primer name	Primer Sequence (5'→3')
AT3G13610	F6'H1_GSP_F	CCTGTTGCTGTGGAAGAGAAG
AT3G13610	F6'H1_GSP_R	GCAGATATCAGGCCAGAACTG
AT3G12900	S8H_GSP_F	CGGTAGCCAAGCGTTAAGTAC
AT3G12900	S8H_GSP_R	CCACCTGTCATTTTCATTTCG
AT4G31940	CYP82C4_GSP_F	TTGTTCCAATCCTTGTTTTCG
AT4G31940	CYP82C4_GSP_R	TATGACCCAAGTGCGTCTCTC
	LBb1.3_SALKTDNA	ATTTTGCCGATTTCGGAAC
	Spm32-R	TACGAATAAGAGCGTCCATTTTAGAGTGA
**Primers used for cloning**		
Transgenic line	Primer name	Primer sequence (5'→3')
proF6'H1:F6'H1-GFP	ggF6'H1pro_For	AACAGGTCTCAACCTCACGAATTCATAACA GATTCACA
	ggF6'H1pro_Rev	AACAGGTCTCATGTTTGGAATAAAAAAGAT AGGAG
	ggF6'H1orf_For	AACAGGTCTCAGGCTAAATGGCTCCAACA CTCTTGAC
	ggF6'H1orf_Rev	AACAGGTCTCACTGAGATCTTGGCGTAATC GAC
proS8H:S8H-GFP	ggS8Hpro_For	AACAGGTCTCAACCTGCAGAACCGAAATTA GTACCG
	ggS8Hpro_Rev	AACAGGTCTCATGTTTCTCCACACTTCTGC TTGAAAA
	ggS8Horf_For	AACAGGTCTCAGGCTATGGGTATCAATTTC GAGGACCA
	ggS8Horf_Rev	AACAGGTCTCACTGACTCGGCACGTGCGA AGTC
proCYP82C4:CYP82C4 -GFP	ggCYP82C4pro_For	AACAGGTCTCAACCTATTCAAGAAAGCAGA GAGATTAGT
	ggCYP82C4pro_Rev	AACAGGTCTCATGTTAGTGTTTTGAGAGTG CAGAAGAGA
	ggCYP82C4cds_For1	AACAGGTCTCAGGCTAAATGGATACTTCCC TCTTTTCTTTG
	ggCYP82C4cds_Rev1	AACAGGTCTCACTGACACAAAAAGTTCTTC CTTAATACGTG
**Primers used for RT-qPCR analysis**		
AT3G18780	ACTIN2_qPCR_F	GACCAGCTCTTCCATCGAGAA
AT3G18780	ACTIN2_qPCR_R	CAAACGAGGGCTGGAACAAG
AT4G05320	UBQ10_qPCR_F	CTTCGTCAAGACTTTGACCG
AT4G05320	UBQ10_qPCR_R	CTTCTTAAGCATAACAGAGACGAG
AT3G13610	F6'H1_qPCR_F	TGATATCTGCAGGAATGAAACG
AT3G13610	F6'H1_qPCR_R	GGGTAGTAGTTAAGGTTGACTC
AT3G12900	S8H_qPCR _F	GGCACCAAATCCCTCCCAGA

AT3G12900	S8H_qPCR_R	TTTTGCCGTCGTGTGGTTGG
AT4G31940	CYP82C4_qPCR_F	TGTGGTTCAAGAATGGCGGT
AT4G31940	CYP82C4_qPCR_R	TCCGACGATACTGAGCCTCC
AT3G53480	PDR9_qPCR_F	ATCTACTCGGCTTGGCTTCG
AT3G53480	PDR9_qPCR_R	CGGTGACTCCCACCAATGAA
AT2G28160	FIT_qPCR_F	GCGGTATCAATCCTCCTGCT
AT2G28160	FIT_qPCR_R	GATGGAGCACCTTCTCCT
AT1G56160	MYB72_qPCR_F	AGTGGTCAAAGATCGCGTCC
AT1G56160	MYB72_qPCR_R	TGTGCTTTGGTCATGAGTGC

Supplemental Table S2. Settings used for the analysis of different mineral elements with sector-field ICP-MS in this study. Supports Material and Methods.

Resolution	Isotope	Accurate mass	Method mass offset	Mass window	Mass range	Settling time	Sample time	Samples per peak	Segment duration	Search window	Integration window	Scan type	Detection mode	Integration type	IS index	IS name	Regression type	Acqu points	Peak shift
Low	B11	11.0088	0.0034	125	10.986 - 11.032	0.300	0.0700	20	1.75	80	60	EScan	both	Average	3	Rh103	Linear	10	1.0
Low	Mo98	97.9049	-0.0199	125	87.729 - 88.081	0.111	0.0200	20	0.480	80	60	EScan	both	Average	3	Rh103	Linear	10	1.0
Low	Rh103	102.9050	-0.0253	125	102.691 - 103.119	0.001	0.0100	20	0.25	80	60	EScan	both	Average	-	-	Linear	10	1.0
Medium	P31	30.9732	-0.0053	135	30.968 - 30.978	0.039	0.0050	20	0.135	60	60	EScan	both	Average	8	Rh103	Linear	10	1.0
Medium	Ca44	43.9549	-0.0082	135	43.948 - 43.962	0.043	0.0050	20	0.135	60	60	EScan	both	Average	8	Rh103	Linear	10	1.0
Medium	Mn55	54.9375	-0.0107	135	54.925 - 54.946	0.038	0.0400	20	1.080	65	60	EScan	both	Average	8	Rh103	Linear	10	1.0
Medium	Fe56	55.9344	-0.0109	135	55.925 - 55.944	0.001	0.0400	20	1.080	60	60	EScan	both	Average	8	Rh103	Linear	10	1.0
Medium	Ni60	59.9302	-0.0114	135	59.919 - 55.941	0.001	0.0600	20	1.080	50	60	EScan	both	Average	8	Rh103	Linear	10	1.0
Medium	Cu63	62.9291	-0.0119	135	62.918 - 62.940	0.001	0.0600	20	1.620	90	60	EScan	both	Average	8	Rh103	Linear	10	1.0
Medium	Zn66	65.9255	-0.0125	135	65.914 - 65.937	0.036	0.0500	20	1.350	50	60	EScan	both	Average	8	Rh103	Linear	10	1.0
Medium	Rh103	102.9050	-0.0182	135	102.888 - 102.922	0.055	0.0200	20	0.540	60	60	EScan	both	Average	-	-	Linear	10	1.0
Medium	Na23	22.9892	-0.0053	135	22.985 - 22.993	0.300	0.0050	20	0.135	60	60	EScan	both	Average	8	Rh103	Linear	10	1.0
Medium	Mg26	25.9821	-0.0061	135	25.978 - 25.986	0.001	0.0100	20	0.270	60	60	EScan	both	Average	8	Rh103	Linear	10	1.0
Medium	S34	33.9673	-0.0067	135	31.967 - 31.977	0.001	0.0100	20	0.270	60	60	EScan	both	Average	8	Rh103	Linear	10	1.0
High	Ge72	71.9215	-0.0127	150	71.917 - 71.926	0.061	0.0500	20	1.500	60	50	EScan	both	Average	-	-	Linear	10	1.0
High	K39	38.9632	-0.0013	140	38.961 - 38.966	0.300	0.1000	20	0.280	50	60	EScan	both	Average	1	Ge72	Linear	10	1.0

Supplemental Table S3. MRM transitions and retention times of coumarins analyzed in this study. Supports Material and Methods.

Compound	Retention time (min)	Precursor ion (m/z)	Product ion (m/z)	Ion mode
**Triple Quad**				
4-Methyldaphnetin	4.72	193.0	147.0	Positive
4-Methyldaphnetin	5.26	191.0	145.0	Negative
Esculin	3.02	341.0	179.0	Positive
Esculetin	3.86	179.0	123.0	Positive
Fraxin	3.53	369.0	207.0	Negative
Fraxetin	4.16	209.0	149.0	Positive
Scopolin	3.38	353.0	191.0	Negative
Scopoletin	4.85	193.0	133.0	Positive
Sideretin (oxidized)	4.40	221.0	177.9	Negative
**Orbitrap**				
Compound	Retention time (min)	Precursor ion (m/z)	Product ion (m/z)	Ion mode
4-Methyldaphnetin	5.99	193.04954	-	Positive
Esculin	3.42	341.0867	-	Positive
Esculetin	4.58	179.0339	-	Positive
Fraxin	4.32	369.0827	-	Negative
Fraxetin	5.14	209.0444	-	Positive
Scopolin	4.02	355.10236	-	Positive
Scopoletin	6.12	193.0495	-	Positive
Sideretin (oxidized)	4.88	221.0916	-	Negative
Sideretin (reduced)	4.40	223.02481	-	Negative
